# Dual Function of iPSC-Derived Pericyte-Like Cells in Vascularization and Fibrosis-Related Cardiac Tissue Remodeling In Vitro

**DOI:** 10.3390/ijms21238947

**Published:** 2020-11-25

**Authors:** Monika Szepes, Anna Melchert, Julia Dahlmann, Jan Hegermann, Christopher Werlein, Danny Jonigk, Axel Haverich, Ulrich Martin, Ruth Olmer, Ina Gruh

**Affiliations:** 1 Leibniz Research Laboratories for Biotechnology and Artificial Organs (LEBAO), Department of Cardiothoracic, Transplantation and Vascular Surgery, Hannover Medical School, 30625 Hannover, Germany; Szepes.Monika@mh-hannover.de (M.S.); Melchert.Anna@mh-hannover.de (A.M.); Dahlmann.Julia@mh-hannover.de (J.D.); Haverich.Axel@mh-hannover.de (A.H.); Martin.Ulrich@mh-hannover.de (U.M.); Olmer.Ruth@mh-hannover.de (R.O.); 2 REBIRTH—Research Center for Translational Regenerative Medicine, Hannover Medical School, 30625 Hannover, Germany; Jonigk.Danny@mh-hannover.de; 3 Biomedical Research in Endstage and Obstructive Lung Disease Hannover (BREATH), Member of the German Center for Lung Research (DZL), Hannover Medical School, 30625 Hannover, Germany; Hegermann.Jan@mh-hannover.de; 4 Institute of Functional and Applied Anatomy, Research Core Unit Electron Microscopy, Hannover Medical School, 30625 Hannover, Germany; 5 Institute of Pathology, Hannover Medical School, 30625 Hannover, Germany; Werlein.Christopher@mh-hannover.de

**Keywords:** cardiac tissue engineering, iPSC-derived vascular cells, pericytes, cardiac fibroblast, myocardial interstitial fibrosis

## Abstract

Myocardial interstitial fibrosis (MIF) is characterized by excessive extracellular matrix (ECM) deposition, increased myocardial stiffness, functional weakening, and compensatory cardiomyocyte (CM) hypertrophy. Fibroblasts (Fbs) are considered the principal source of ECM, but the contribution of perivascular cells, including pericytes (PCs), has gained attention, since MIF develops primarily around small vessels. The pathogenesis of MIF is difficult to study in humans because of the pleiotropy of mutually influencing pathomechanisms, unpredictable side effects, and the lack of available patient samples. Human pluripotent stem cells (hPSCs) offer the unique opportunity for the de novo formation of bioartificial cardiac tissue (BCT) using a variety of different cardiovascular cell types to model aspects of MIF pathogenesis in vitro. Here, we have optimized a protocol for the derivation of hPSC-derived PC-like cells (iPSC-PCs) and present a BCT in vitro model of MIF that shows their central influence on interstitial collagen deposition and myocardial tissue stiffening. This model was used to study the interplay of different cell types—i.e., hPSC-derived CMs, endothelial cells (ECs), and iPSC-PCs or primary Fbs, respectively. While iPSC-PCs improved the sarcomere structure and supported vascularization in a PC-like fashion, the functional and histological parameters of BCTs revealed EC- and PC-mediated effects on fibrosis-related cardiac tissue remodeling.

## 1. Introduction

Myocardial interstitial fibrosis (MIF) has been described as a pathophysiological process characterized by increased tissue stiffness, reflecting excessive extracellular matrix (ECM) deposition and the progressive loss of parenchymal cells, which impairs organ function [1]. While “activated fibroblasts (Fbs)” or “myofibroblasts” have been considered the principal source of ECM for many years, a contribution of perivascular cells has been described recently for various organs, including pericytes (PCs) in kidney fibrosis [2], hepatic stellate cells (a liver-specific pericyte population) in liver fibrosis [3], and lung PCs in pulmonary fibrosis [4]. For the heart, human coronary microvascular PCs have been shown to secrete ECM proteins and cytokines [5,6], but PC’s contribution to MIF remains unclear (reviewed in [7]).

Pericytes are known to regulate endothelial function, vessel permeability, and stabilization. They can be identified by the co-expression of a set of markers—e.g., neuron/glia-type 2 antigen (NG2), PDGFRβ-receptor, CD73, CD90, CD105, CD146, and CNN1 [8,9,10]. In native tissue, PCs are mainly found in the microvasculature, while vascular smooth muscle cells (vSMCs) sharing a common origin and displaying a considerable phenotypic overlap with PCs are located in larger vessels [11,12,13]. The in vitro specification of pericytes can be achieved by the differentiation of hemangioma stem cells [8] or pluripotent stem cells, yielding CD105^+^/CD90^+^/CD73^+^/CD31^−^ multipotent mesodermal precursors [9] or via a bipotential early vascular cell (EVCs) population which can mature into endothelial cells (ECs) and PCs and self-organize to form microvascular networks [14]. The directed differentiation of ECs and PCs from human pluripotent stem cells (hPSCs) via mesoderm induction, vascular specification, and cell sorting generated 50–60% CD31^−^ PCs, which supported vascular network formation in an EC-PC co-culture model [15].

Due to the shortage of available patient samples and interspecies variations in nonhuman cardiomyocytes or animal models [16], new tools are needed to obtain a more detailed understanding of the role of PCs in cardiac fibrosis. Three-dimensional in vitro models of the human heart, such as bioartificial cardiac tissues (BCTs), offer the unique opportunity to study excessive ECM production, abnormal myocardial stiffening, and the individual contribution of the cell types involved (i.e., cardiomyocytes, ECs, Fbs, PCs, and vSMCs). In this context, induced pluripotent stem cells (iPSCs) allow for the directed de novo formation of all contributing cardiovascular cell types. In our laboratory, we have previously shown that undifferentiated hPSCs can be expanded in scalable suspension cultures and in stirred tank bioreactors [17] and can be efficiently differentiated towards cardiomyocytes (CMs) [18,19] and ECs [20]. In addition, we developed a customized bioreactor system that is capable of generating, culturing, mechanically stimulating, and evaluating miniaturized BCT constructs consisting of primary [21] or hPSC-derived CMs [22] and different composites of ECM [23]. The BCT model allows for the modulation and investigation of tissue stiffness and the application of preload in vitro [22] and can be used as a cardiac disease model [24,25].

While endothelium-mediated contributions to fibrosis have been described (reviewed in [26]), ECs have not been included in current in vitro models of cardiac fibrosis yet. Most of them focus on the role of cardiac Fbs in combination with rat primary ventricular cardiomyocytes in engineered 3D cardiac fibrotic tissue [27] or human iPSC-CMs in 3D microtissues [28]. Cardiac fibroblasts are suggested to share a common developmental origin with SMCs and PCs during cardiogenesis (i.e., epicardium, neural crest) [29], and additionally show a signature of cell surface markers that has previously been described for mesenchymal stem cells [30]. For other organs, fibrosis models based on human iPSCs also included pericyte-like cells—i.e., iPSC-derived hepatic stellate cells for the in vitro modelling of liver fibrosis [31]—or mesangial cells and pericytes in kidney organoids from iPSCs [32].

In this study, we established a differentiation and selection protocol for high-purity pericyte-like cells from human iPSCs (iPSC-PCs). The gene expression pattern of the derived iPSC-PCs was compared with that of primary PCs and the angiogenic potential was examined in functional assays, such as EC/PC co-cultures. Subsequently, the differentiated iPSC-PCs in combination with purified hPSC-CMs and ECs were used in the established model of BCT formation to investigate the role of PC-like cells in the tissue morphology, organization, electrical properties, and in endothelial vascular network formation in comparison to fibroblast addition. In this model, we revealed a substantial contribution of iPSC-PCs to tissue remodeling, impacting tissue mechanics and function in vitro, thereby representing a novel complex 3D in vitro model for cardiac tissue fibrosis in vivo.

## 2. Results and Discussion

### 2.1. Pericyte-Like Cells Can Be Generated by In Vitro Differentiation of iPSCs

**Figure 1:**
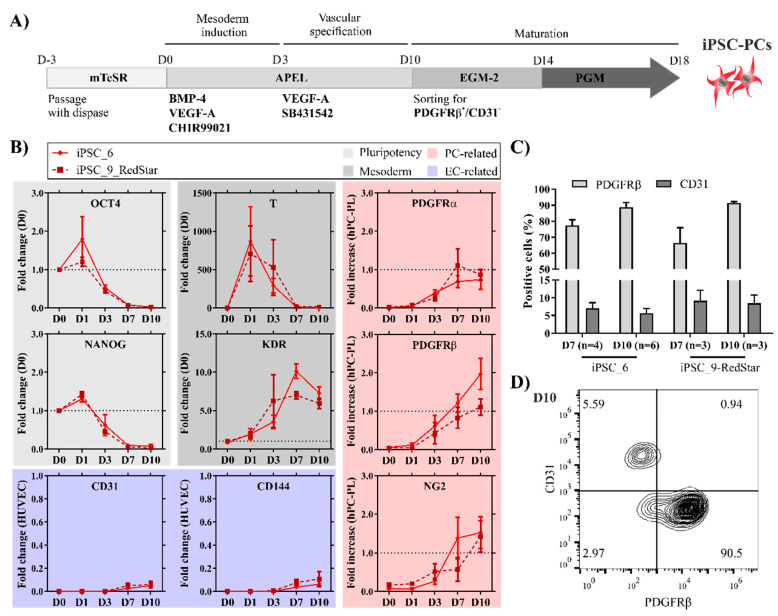
PC-like cells can be efficiently generated from human iPSCs. (**A**) Schematic representation of the differentiation process depicting the timeline as well as the used small molecules, growth factors, and media. (**B**) Pluripotency markers OCT4 and NANOG gradually decrease relative to the D0 of differentiation determined by qRT-PCR. Upregulation of early mesodermal marker T, followed by the upregulation of Kinase Insert Domain Receptor (KDR), indicates the induction of mesoderm. Expression levels of PC markers (PDGFRα, PDGFRβ, and NG2) are expressed relative to human placental pericytes (hPC-PLs), and the EC markers (CD31 and CD144) are shown relative to HUVECs [35] (*n* = 3–6). (**C**) Flow cytometric analysis for CD31 and PDGFRβ on D7 and D10 in both iPSC lines used for differentiation (*n* = 3–6). (**D**) Representative plot of stained (CD31, PDGFRβ) iPSC6-derived cells on differentiation D10 prior to fluorescence-activated cell sorting.

The differentiation process modified from Orlova et al. [15,33] consists of a mesoderm induction phase using a high BMP4 concentration combined with VEGF-A and the activation of the Wnt-pathway (CHIR99021), followed by a vascular specification phase with continued VEGF-A application and TGFβ inhibition (SB431542), cell sorting, and further maturation (Figure 1A).

Upon mesoderm induction, the colonies of iPSCs increased in size with migrating cells after D2, and cells with a cobble stone-like morphology and spindle-like mesenchymal morphology, resembling ECs and PCs, were identified from D3 onwards (Figure S1A). Successful mesoderm induction was indicated by the rapid upregulation and subsequent downregulation of the early mesodermal marker T-Brachyury (T), followed by upregulation of the lateral plate mesoderm marker KDR (Figure 1B). The expression of KDR together with PDGFRα marks cardiovascular progenitors that can further differentiate into CMs, ECs, and mural cells [34].

Accordingly, from D3 onwards, the expression levels of the PC markers NG2, PDGFRα, and PDGFRβ, as well as the expression level of the EC markers, CD31 and CD144, increased. Meanwhile, a marked decrease in the pluripotency-associated genes OCT4 and NANOG expression was observed after 3 days. On D7, around 70% of all cells were positive for PDGFRβ for both used cell lines (Figure 1C). The expression increased until D10, resulting in 88.8 ± 2.98% and 91.5 ± 0.82% PDGFRβ+ cells for the iPSC6 and the iPSC9_RedStar cells, respectively, while the CD31+ endothelial cell population remained at <10%. As there was little to no co-expression of CD31 and PDGFRβ, we conclude that the cells adopted either an EC or pericyte-like phenotype (Figure 1D).

Thus far, PCs were generally differentiated from hPSCs mainly as a side product of endothelial differentiation and not as the main target population of the differentiation. Masumoto et al. [36] recently published the generation of 74.4 ± 8.4% PDGFRβ^+^ mural cells (MC) after differentiation induced by BMP4 and Wnt activation. However, the resulting cells were not characterized in detail. The modifications described and applied in this study improved the differentiation towards the enrichment of around 90% PDGFRβ+ pericytes in both tested cell lines, which were then further separated from other cell types by positive selection and extensively analyzed for their functional and molecular characteristics (see below).

### 2.2. Highly Purified Human iPSC-PCs Support the Generation of Endothelial Tube-Like Structures In Vitro

In contrast to other protocols for PC purification, which depend on the negative selection (removal) of CD31^+^ cells [9,15], we applied an antibody-based positive selection of PDGFRβ, which resulted in a 99% purity, while the potential risk for the remaining (CD31^−^/PDGFRβ^−^) pluripotent cells or other cell lines was minimized. The sorted cells proliferated in culture for up to seven passages, with a slower expansion from passage 4 onward, and could be cryopreserved without changes in morphology and marker expression.

**Figure 2:**
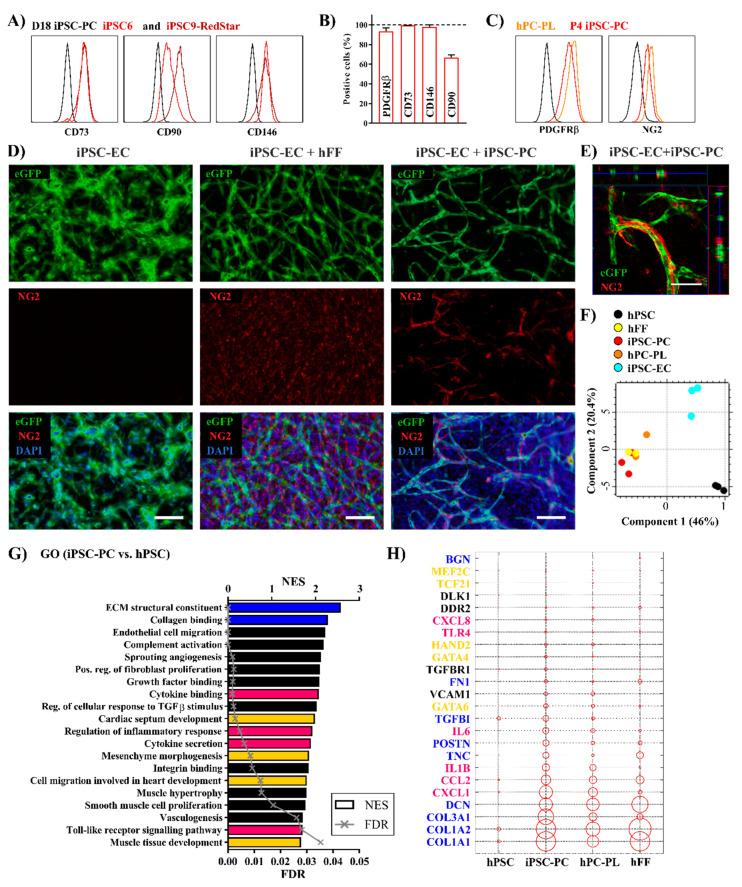
iPSC-PCs exhibit a PC-like function and cardiac phenotype. (**A**) Flow cytometric analysis of the mesenchymal surface marker expression post-maturation on D18 (black line—isotype control) and, (**B**) following expansion, in P4 (*n* = 3). (**C**) The level of PDGFRβ and NG2 in iPSC-PCs (P4) compared to hPC-PL (black line—isotype control). (**D**) Network formation in fibrin matrices (cultured in EGM-2 for 7 days) containing iPSC-EC only, iPSC-EC+hFF, and iPSC-EC+iPSC-PC, and NG2 expression in the fixed co-cultures. Nuclei stained with DAPI, iPSC-ECs express eGFP (iPSC9_eGFP). Scale bars: 100 µm. (**E**) Higher magnification Z-stack image of iPSC-EC+iPSC-PC networks with orthogonal projections. (**F**) PCA plot showing the first two principal components (data points represent one sample in the microarray analysis). (**G**) Significantly enriched gene ontologies (GOs) in iPSC-PC vs. hPSC (selected genes from the GOs marked with blue, pink, and yellow are shown on plot G). (**H**) Expression of ECM-related structural genes (blue), cardiac transcription factors (yellow), and immunomodulatory cytokines/receptors (pink) in hPSCs, primary PCs and Fbs, and iPSC-PCs. On the balloon plot, the size of the circles represents the average gene expression of sample groups relative to GAPDH (scaling factor: 300×).

The mesenchymal stem cell markers CD73 and CD90 as well as the pericyte markers CD146 and PDGFRβ (Figure 2A,B and Figure S1B) showed a strong expression, both on D18 and after four passages. The expression levels were comparable not only to primary PCs (hPC-PL), but also to human foreskin fibroblasts (hFF) (Figure S1B), whereas endothelial markers were absent in all cell types. The presence of NG2 was confirmed both by IF and flow cytometry (Figure S1C and Figure 2C). Immunofluorescent (IF) staining for the structural proteins vimentin (VIM), alpha smooth muscle actin (αSMA), and calponin (CNN1) revealed a high similarity between the pericyte growth medium (PGM) cultivated cells and hPC-PLs, both showing a heterogenic expression of said smooth muscle-related contractile proteins (Figure S1C). Upon maturation, mesenchymal surface markers and PC-like phenotype-related contractile proteins were preserved over several passages as well as the PDGFRβ expression and proliferation ability, which is highly similar to the characteristics of D17 mesenchymoangioblast-derived hPCS-PCs described by Kumar et al. [37]. In summary, an effective and reproducible differentiation method for the generation of highly purified iPSC-derived PC-like cells was successfully established.

Traditionally, PCs are defined based on their localization in non-muscular microvessels, capillaries, and postcapillary venules, playing a key role in vessel integrity and maturation. PCs are embedded in the microvascular basement membrane, incompletely enveloping the endothelial cells [6]. The consequences of dysfunctional vessel permeability range from neurodegenerative disorders [38], as PC/EC interactions are critical to regulating the blood brain barrier [39], to cardiovascular diseases, because PCs can contribute to plaque microvascularization and stability in atherosclerosis [40]. Thus, we tested the ability of iPSC-PCs to support the angiogenic potential of ECs in a 3D in vitro EC-PC co-culture model in a fibrin gel [41]. ECs were differentiated as recently published [20,42] (Figure S2E) using the iPSC9_eGFP cell line, which allows for the live monitoring of iPSC-ECs in co-culture assays. Highly purified iPSC-ECs (97% CD31+ cells after magnetic cell separation) expressed typical EC markers (CD31, CD144, and vWF) (Figure S2F).

Consistent with earlier findings [41], iPSC-ECs in monoculture connected with each other but did not form tube-like structures during the 7 days of cultivation (Figure 2D and Figure S3A). When hFFs or iPSC-PCs were added to the ECs in a 3:1 ratio in EGM-2, intensely branched networks formed from day 3 (Figure S3B,C). Immunofluorescence staining revealed that hFFs formed an NG2^+^ feeder layer-like structure but did not wrap around the tubular EC networks (Figure 2D and Figure S3E). As expected, no NG2 expression was detected in the pure iPSC-EC cultures. In contrast, in iPSC-PC containing co-cultures NG2^+^ cells were found closely attached to the formed capillary-like structures (Figure 2D,E and Figure S3E).

In conclusion, within this co-culture model iPSC-PCs displayed a pericyte-like functionality which is supposed to be required to achieve an interconnected EC-network in fibrin gels [43]. However, we did not investigate the further maturation and permeability of the formed networks, an important functional aspect which can differ upon the addition of different mural cell types [44].

### 2.3. Human iPSC-PCs Show Both a Pericyte-Like and Cardiac Fibroblast-Like Gene Expression Profile

For an in-depth characterization of the iPSC-PCs and iPSC-ECs, gene expression profiling was performed using microarray analysis in comparison to their undifferentiated origin, primary PCs and hFFs (as described in Table S1). The principal component scores for the iPSC-PCs and iPSC-ECs, respectively, were distinct from the undifferentiated iPSCs, indicating successful differentiation. At the same time, similar scores within groups of individual differentiation experiments demonstrated the reproducibility of the applied protocols. Moreover, the iPSC-PCs clustered together with the primary PCs and Fbs as well (Figure 2F). There were 4806 differentially expressed genes (DEGs) in the transcriptome of iPSC-PCs when compared to hPSCs, and 3240 DEGs compared to iPSC-ECs. The gene expression pattern was similar between the iPSC-PCs and hFFs as well as the hPC-PLs (785 and 22 DEGs, respectively) (Figure S4A). Interestingly, there was a remarkably large difference regarding the gene expression pattern in between the two tested commercially available hPC-PL isolations (Figure 2F).

The relevant gene ontologies (GOs) significantly upregulated in iPSC-PCs compared to undifferentiated cells include gene sets connected to fibroblast proliferation and ECM structure, such as collagen binding and fibril organization, and the binding of growth factors (Figure 2G). The ability of iPSC-PCs to support vascular structures is also reflected by the GOs “endothelial cell migration” and “sprouting angiogenesis”, thus confirming their characterization as “pericyte-like”. The enriched terms “cytokine binding” and “complement activation” were also in line with the pro-coagulatory and pro-inflammatory nature of PCs [45,46]. Similar to earlier descriptions in primary [47] and differentiated capillary PCs [37], the chemoattractants CXCL1 and CXCL8, inflammatory cytokines IL1B and IL6, and the adhesion molecule VCAM1 were upregulated (Figure 2H) in hPC-PLs and PCs compared to hFFs.

A cardiac mesenchymal origin of iPSC-PCs was indicated by the enrichment of the GOs “mesenchyme morphogenesis”, “cardiac septum development”, and “cell migration in heart development” (Figure 2G). A detailed analysis revealed the expression of the cardiac transcription factors HAND2, MEF2C, and TCF21 in both hPC-PL and iPSC-PCs, whereas GATA4 and POSTN, also known to mark cardiac fibroblasts (CFs), were highly upregulated in iPSC-PCs. Interestingly, CF-related genes were significantly enriched in both hPC-PLs and iPSC-PCs compared to the hFFs of dermal origin (Figure S4B) [30,48]. The structural ECM components such as collagen I (COL1A1 and COL1A2), fibronectin (FN1), lumican (LUM), and decorin (DCN) showed a higher similarity in expression patterns across the hFFs and iPSC-PCs (Figure 2H). Biglycan (BGN), known for its role in infarct healing by ensuring proper collagen scar formation [49], was only strongly expressed in hFFs.

While iPSC-PCs and iPSC-ECs can be derived simultaneously from iPSCs in the same differentiation protocol, microarray analyses confirmed distinct expression profiles for the two cell types. iPSC-ECs upregulated pathways involved in the development of the cardiovascular system, including vasculogenesis, angiogenesis, and heart morphogenesis (Figure S4C). A more detailed analysis showed that endothelial linage specific genes together with genes related to EC function and markers for arterial ECs [20,50] were strongly upregulated (Figure S4D). The results suggest that the differentiated iPSC-ECs express the key regulators of endothelium-related processes.

### 2.4. Replacing Fibroblasts with iPSC-PCs for Cardiac Tissue Formation Leads to a Fibrosis-Like Phenotype

**Figure 3:**
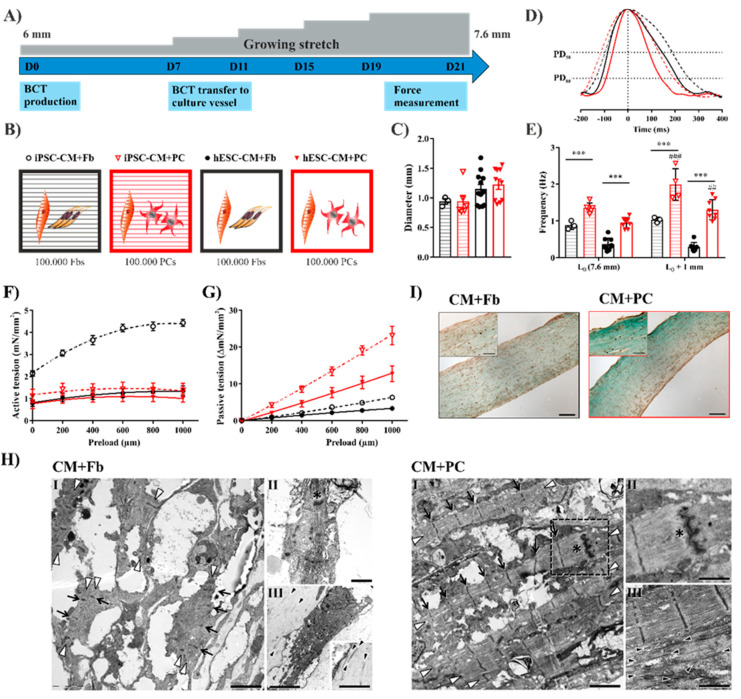
iPSC-PCs in cardiac tissues initiate a fibrosis-like remodeling process. (**A**) Schematic representation of BCT cultivation with growing stretch. (**B**) 1 × 10^6^ iPSC- or hESC-derived CMs were mixed either with 1 × 10^5^ hFFs or 1 × 10^5^ iPSC-PCs for tissue formation. The colors and symbols serve as legends for the other diagrams of the illustration. (**C**) Tissue diameter measured on D21 (for **C** and **E**–**G** BCT with iPSC-CM *n* = 3–6; BCT with hESC-CM *n* = 9–12 tissues per group) (**D**) Representative spontaneous contractions normalized to peak height (iPSC-CMs—dashed lines, hESC-CMs. continuous lines). PD—peak duration at 50% and 80% peak height. (**E**) Frequency of spontaneous contraction before and after force measurement (*** *p* < 0.001 between indicated groups; ^##^ *p* < 0.01 and ^###^ *p* < 0.001 L_0_ vs. L_0_ + 1 mm). (**F**) Force of paced isometric contractions recorded over increasing preload measured on day 21. (**G**) Passive tension development with increasing preload. (**H**) Transmission Electron Microscopy of longitudinally sectioned BCTs. Left: hESC-CM+Fb; right: hESC-CM+PC. I: Myofibrils (white arrowheads) are visible in both tissues, however are more regularly distributed in CM+PC, with a higher width and constant Z-lines (arrows), while the fibrils in CM+Fb are narrow and the Z-lines appear out of phase. II: Cell–cell contacts (asterisks, right: enlargement from boxed area in I) are visible in both tissues. III: Collagen fibrils (black arrowheads) are dispersed in CM+Fb, while they are regularly arranged in CM+PC. Scale bars: I, 2 µm; II, 1 µm; III left, 5 µm/inset 1 µm; III right, 1 µm. (**I**) Masson’s trichrome staining of 7 µm paraffin sections of iPSC-CM BCTs with Fb (left) and iPSC-PC (right) image (scale bar: 100 µm).

Earlier experiments demonstrated the necessity of fibroblasts together with CMs for functional cardiac tissue formation [22], but the tissue remodeling capacity of PC-like cells was not investigated before. Therefore, we addressed if our differentiated iPSC-PCs could replace the conventionally used dermal fibroblasts in bioartificial cardiac tissues with CMs differentiated either from hESCs or from iPSCs using our previously established protocols [19,22] (Figure 3A). The directed cardiac differentiation of human PSCs resulted in aggregates contracting from D7 onwards with 95.6 ± 1.5% cTnT^+^ cardiomyocytes following the antibiotic selection of transgenic (α-MHC^p^ Neo^R^) cells [22,51] (Figure S2A–D). There is a huge debate on the number of PCs in the human heart; while some groups reported PCs as being the second most abundant cell type [52], others determined that PCs occupy only 5% of the non-myocyte fraction [53]. We chose the addition of 10% Fbs or iPSC-PCs, respectively (Figure 3B), as a ratio of 10:1 for CMs:Fbs was proven to be optimal for tissue formation in earlier experiments by our group and others [19,22,54].

In all the tested conditions simultaneously contracting, organized tissues were formed showing a similar macroscopic morphology (Figure S5A,B) and comparable final tissue diameter (Figure 3C). Dermal Fbs and iPSC-PCs were able to contract the collagen-Matrigel hydrogels, resulting in the remodeling and complete consolidation of the tissues both with iPSC-CMs and hESC-CMs after 21 days. The cell distribution and longitudinal alignment within the tissues was comparable based on immunofluorescence staining for cTnT and VIM, followed by quantitative image analysis [55] (Figure S5A,B).

The mechanical and electrical properties of the tissues were analyzed after 3 weeks of cultivation (D21) in a custom-made multimodal bioreactor system [21], as already described [19]. The spontaneous contraction peaks were asymmetric in all tissues (just like in healthy myocardium), showing a steep activation and a prolonged relaxation phase (Figure 3D). The relaxation time and overall duration of contractions was significantly lower in both iPSC-PC-containing groups (Figure S5D). While there was a constant and significantly lower spontaneous beating rate in the CM+Fb BCTs, the rate was higher in the CM+PC groups and it increased further with increasing preload (Figure 3E). This stretch-induced frequency increase may recapitulate the myocardial stress response, which acts as a compensatory mechanism once the optimal sarcomere length is reached.

Isometric contraction forces were recorded in response to electrical stimuli, while the preload was increased stepwise in 200 µm increments (Figure 3F). All the BCT groups reacted according to the Frank–Starling mechanism, thus an increase in the preload led to increased contraction forces until the sarcomere length with the maximum contraction force (L_max_) was reached. Active forces showed the highest value in the iPSC-CM+Fb BCTs (4.43 ± 0.15 mN/mm^2^; hESC-CM+Fb: 1.36 ± 0.22 mN/mm^2^; iPSC-CM+PC: 1.49 ± 0.29 mN/mm^2^; and hESC-CM+PC: 1.10 ± 0.21 mN/mm^2^) as well as the greatest increase in active force production with increasing strain indicating the greatest contractility (Figure S5F). In both the iPSC-PC-containing groups, the L_max_ was reached on average at a 600–800 µm preload, indicating a longer initial sarcomere length, while in Fb-containing BCTs the contraction force increased until a 1000 µm preload (Figure S5C). A decrease in the contractility and active force generation are typical (but not exclusive) processes in tissues with MIF, therefore in this context they have to be discussed relative to the tissue stiffness.

Interestingly, the passive forces (Figure 3G), indicating the stiffness of the tissues, were clearly higher in the BCTs where the Fbs were replaced by iPSC-PCs, which would correspond to a less compliant tissue. The elastic modulus, calculated from the linear elastic region of the stress–strain curve (Figure S5G) was in the physiological range for BCTs with Fbs (iPSC-CM+Fb: 31.35 ± 2.41 kPa and hESC-CM+Fb: 21.54 ± 3.91 kPa); the addition of iPSC-PCs, however, resulted in a tissue stiffness similar to that of fibrotic hearts [56] (iPSC-CM+PC: 128.10 ± 13.53 kPa and hESC-CM+PC: 74.91 ± 13.59 kPa). The pathological levels of tissue stiffness could be the cause for the lower active forces measured in PC-containing tissues, since more contractile work is needed to induce the same displacement [57].

The post-rest potentiation (PRP), a measure of the capacity of the sarcoplasmic reticulum to store and release Ca^2+^, was less pronounced in hESC-CM+PC BCTs (Figure S5E). This effect on PRP was also observed in a tissue model for mimicking interstitial cardiac fibrosis [58] together with decreased active contraction forces and increased tissue stiffness.

In line with the increased passive forces, we observed excessive collagen deposition and a high organization towards the longitudinal axis in iPSC-PC-containing BCTs (Figure 3H), a third hallmark of a fibrotic phenotype. Moreover, tissues with iPSC-PCs exhibited a higher organization of the sarcomeres with the regular distribution of Z-bands, and H-bands were also visible in some cases.

Elevated ECM deposition was also confirmed in the BCTs prepared with iPSC-PCs by a strong blue color in Masson’s trichrome staining (Figure 3I) and by quantification of the collagen content in Gömöri’s trichrome staining (Figure S5H) [59,60]. The presence of a basal lamina, though incomplete, and formation of intercalated discs with gap junctions between CMs, was proven on high-magnification electron microscopic images in all groups (Figure 3H).

To compare these results with the existing models of TGFβ-induced cardiac fibrosis [28,45], we stimulated hESC-CM+Fb BCTs with 5 ng/mL TGFβ1 for 7 days (D7–D14) and found on average decreased contractility and higher passive forces than without TGFβ1 (Figure S5I–M). However, the effects were not significant and were markedly lower than in BCTs with iPSC-PCs.

### 2.5. Fibrosis-Related Cardiac Tissue Remodeling Is More Pronounced in a Tri-Culture Model with ECs

Endothelial cells (ECs) have been included in myocardial tissue engineered constructs with the aim of in vitro vascularization; together with stromal cells, they improved the hPSC-derived tissue formation and vascular network growth in matrix-free scaffolds [61] and in a fibronectin-gelatine matrix [62].

**Figure 4:**
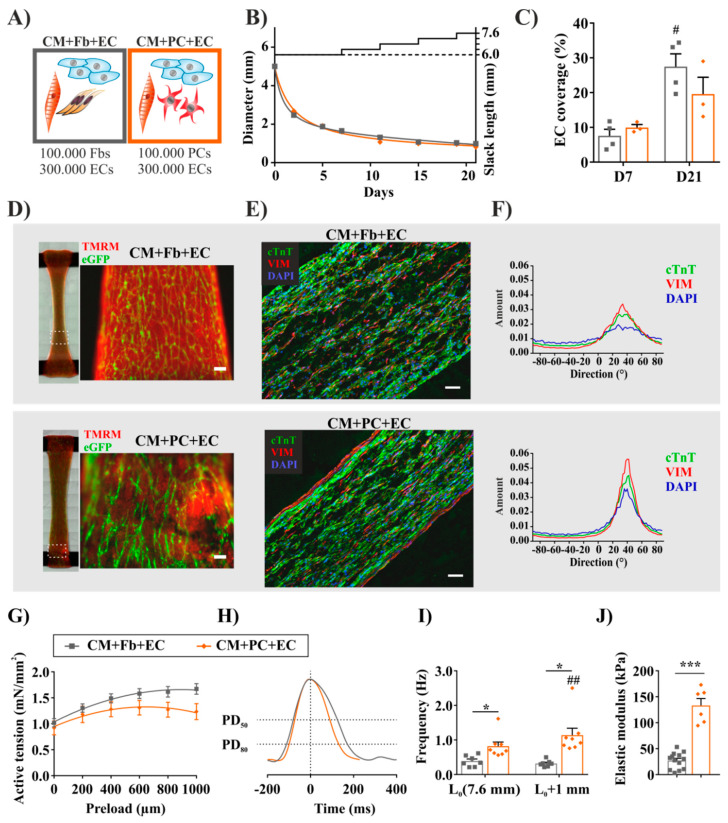
Fibrosis-related cardiac remodeling in a tri-culture model with ECs. (**A**) Cell compositions used for tissue preparation. All the BCTs contained 1 × 10^6^ CMs, plus the indicated amount of other cell types. The different colors of the frames serve as legends for the other diagrams of the illustration. (**B**) Monitoring of tissue remodeling indicated by diameter change over time (*n* = 3–4). (**C**) Development of EC networks during cultivation. EC coverage expressed as the percentage of surface area (^#^ *p* < 0.01 vs. D7). (**D**) Live imaging of the BCT morphology on D21. CMs are visualized by tetramethylrhodamin-methylester (TMRM, selectively labeling viable mitochondria-rich cells), ECs express endogenous eGFP (scale bar: 100 µm). (**E**) Immunofluorescent staining for cTnT and VIM to observe cell distribution, nuclei stained by DAPI (scale bar: 100 µm). (**F**) Directionality analysis of the tissues stained in sub-figure E. The *Y*-axis label “Amount” represents the proportion of structures with the dominant orientation. (**G**) Frank–Starling curves recorded on D21. (**H**) Representative spontaneous contractions normalized to peak height. PD—peak duration at 50% and 80% peak height. (**I**) Frequency of spontaneous contractions before and after force measurement at 0 and 1 mm preload (^##^ *p* < 0.01 vs. L_0_). (**J**) Calculated elastic modulus (**B** and **G**–**J**: *n* = 6–12 BCTs per group, * *p* < 0.05 *** *p* < 0.001 between indicated groups).

Since ECs and PCs are closely connected and have an important interplay in vivo, we extended our previously established two cell type co-culture model with the addition of iPSC-ECs. To this end, 3 × 10^5^ iPSC-ECs per tissue were combined with cardiomyocytes and with either dermal Fbs (CM+Fb+EC) or iPSC-PCs (CM+PC+EC) (Figure 4A). Both cell mixtures formed spontaneously contracting tissues macroscopically similar to each other as well as to their EC-free counterparts, with respect to tissue remodeling (Figure 4B), morphology, and overall cell distribution (Figure 4D). EC-networks typically started forming already on D1, reached a homogeneous distribution throughout the whole tissue during the first seven days of culture, and remained stable over cultivation while the density increased (Figure 4C,D).

After we thus confirmed the feasibility, morphological changes, and alignment of ECs in our 3D model, we focused on the morphological differences introduced by PCs. In line with the observations in EC-free groups (Figure 3H), the effect of iPSC-PC addition on tissue organization towards the longitudinal axis was more pronounced then the effect of Fbs. CM+PC+EC BCTs demonstrated a higher degree of longitudinal alignment [55] in a quantitative image analysis after immunofluorescence staining for cTnT and VIM (Figure 4E,F). Although the final tissue diameter did not differ based on the cell composition, BCTs with iPSC-PCs exhibited a more dense inner structure and a VIM^+^ outer layer. VIM^+^ iPSC-PCs exhibited a more elongated morphology, and thereby a higher cell aspect ratio than Fbs. Similar morphological changes were experienced by Seo et al. when seeding stromal cells in collagen matrices with increased stiffness [63].

Concerning contractile function, active tension-preload relationships for both cell compositions were consistent, with a positive Frank–Starling relationship (Figure 4G). Again, the maximum contraction force was reached at lower preload values in the CM+PC+EC group (Figure S6A) and, on average, BCTs exhibited higher maximum active forces when Fbs (1.68 ± 0.10 mN/mm^2^) were added (CM+EC+PCs: 1.34 ± 0.14 mN/mm^2^) (Figure S6B). Notably, as for the EC-free tissues, iPSC-PCs were responsible for alterations in electrophysiological properties compared to fibroblasts, such as shorter relaxation time and contraction durations (Figure 4H and Figure S6C), and an increase in spontaneous frequency at higher preloads (Figure 4I). Nevertheless, the hallmarks of mature myocardial function—e.g., post-rest potentiation (Figure S6D) and the frequency-dependent acceleration of relaxation (Figure S6E)—were demonstrated for all tissues. Interestingly, the differences in PRP between Fb and PC-containing BCTs were diminished when ECs were added as well.

Finally, with respect to fibrosis-related cardiac tissue remodeling, we investigated the influence of ECs on the PC-mediated tissue stiffness. As in the previous results without ECs, passive tension values (Figure S6F) at the maximum preload were significantly higher in tissues containing PCs. Elastic moduli (Figure 4J) were either in a normal physiologic (CM+Fb+EC: 27.13 ± 4.33 kPa) or in the pathologic (CM+PC+EC: 133.10 ± 13.50 kPa) range [56,57]. These values were significantly higher than for the respective EC-free group (hESC-CM+PC: 74.91 ± 13.59 kPa; Figure S5G), although ECs themselves do not contribute to tissue stiffness significantly (data not shown). Masumoto et al. [36] found higher passive forces, stronger myofiber alignment and gel compaction, as well as a lower relaxation time in iPSC-derived cardiac tissues with CMs and PDGFRβ^+^ mural cells with or without ECs (CM+MC and CM+EC+MC) compared to CM+EC tissues, indicating the MCs’ contribution to higher stiffness.

As a first hint on the underlying mechanisms, upregulated collagen I and III synthesis correlated with the measured passive forces, where BCTs with PCs showed a higher expression both in mRNA levels (Figure S6G) and after histological staining for Sirius Red [64] (Figure S6H). Despite the lower active forces measured in the CM+PC+EC tissues, the organization of individual myosin filaments increased with the higher levels of tissue stiffness (Figure S6I). We hypothesize that iPSC-PCs could be further activated in tissues by EC-mediated signaling, leading to increased ECM production and fibrosis-related tissue remodeling.

### 2.6. Tissues with iPSC-PCs Exhibit Molecular Signatures of Activated Fibroblasts Found in Cardiac Fibrosis

To investigate if iPSC-PCs could be activated similarly to fibroblasts in fibrotic tissue and to elaborate how EC addition could have a pro-fibrotic effect, we aimed at attributing molecular differences in the gene expression of tissues with varying cellular composition to the different cell sources.

**Figure 5:**
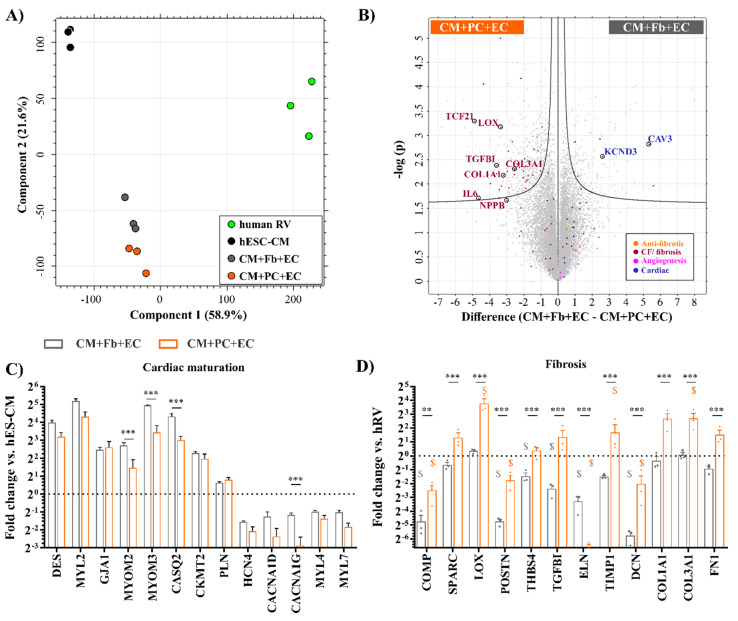
BCTs with iPSC-PCs exhibit the molecular signatures of myocardial interstitial fibrosis. (**A**) PCA plot showing the first two principal components. (**B**) Vulcano plot showing differences in gene expression between CM+PC+EC and CM+Fb+EC BCTs. The areas above the black curves contain significantly upregulated genes on both sides. Expression of cardiac maturation-related genes (**C**) in BCTs normalized to hESC-CMs (all genes in C are significant vs. hESC-CMs) and fibrosis-related (**D**) genes normalized to hRV genes significant vs. hRV are marked by $; ** *p* < 0.01 *** *p* < 0.001 between indicated groups; *n* = 3 in each group. hRV—human right ventricle.

To this end, the transcriptome of the BCT groups (CM+Fb+EC, CM+PC+EC) showing functional and electrophysiological maturation was compared to the hESC-CM source and adult human ventricle (Table S2). We found that BCTs represent an intermediate state between differentiated cardiomyocytes and the adult human ventricle (Figure 5A), according to the first principal component (53.8% of all variance). The BCT groups cluster closer together, which is in line with the number and distribution of DEGs across the analyzed samples (Figure S7A). We chose the source hESC-CMs as the control group to depict differences in the cardiac maturation-related transcripts. Genes related to fibrotic response and vascularization were normalized to the healthy human ventricle (hRV) samples, since they contain all relevant cardiovascular cell types.

Consistent with the observed endothelial network formation, the addition of ECs led to the upregulation of endothelial function-associated transcripts (NOS3) and growth factors (VEGFC, PDGFB) similar to native tissue in both BCT groups. The expression of typical endothelial surface markers and receptors (TEK, NRP1, FLT1) could also be confirmed (Figure S7C). In a direct comparison of the CM+Fb+EC and the CM+PC+EC groups displayed as a Vulcano plot, a balanced distribution of the EC-related transcripts was observed (Figure 5B and Table S3).

While addressing the hallmarks of cardiac maturation [65,66], we found that the embryonic (MYL4) and atrial (MYL7) myosin light chain types were downregulated over CM maturation in BCTs, while the ventricular-type transcript increased (Figure 5C). Ion channels related to pacemaker activity and SA node action potential generation were less expressed in BCTs, whereas an the upregulation of genes involved in Ca^2+^ handling (PLN, CASQ2), energy transduction (CKMT2), and cell–cell interactions (GJA1) was observed. CASQ2 and myomesins (MYOM2 and MYOM3), components of the myofibrillar M band showed a significant increase in the BCTs with Fbs. Similarly, genes associated with cardiac maturation were predominantly located on the right side on the Volcano plot, representing BCTs with Fbs. Interestingly, the ion channel KCND3 and caveolin 3 (CAV3)—an indicator of functional T-tubules—were strongly upregulated (Figure 5B), indicating more mature CMs.

The maturation of the CMs inside the BCT types was also addressed via GSEA, where an adult cardiomyocyte gene set [65] was used to compare BCTs to the freshly differentiated CMs (Figure S7B). As result, the gene set was significantly enriched in both tissue groups, but higher scores were measured in the condition with Fbs.

In line with a fibrotic tissue profile, in BCTs with iPSC-PCs an upregulation in the expression of the ECM proteins (FN1, collagen type I and III, DCN), TGFBI, thrombospondin (THBS4), and periostin (POSTN) was observed (Figure 5D). The collagen-binding proteins COMP and SPARC, which have been associated with fibrotic process [67,68], also showed a significantly higher level in iPSC-PC- vs. Fb-containing BCTs. To better understand why Fb containing tissues fell into the normal range of tissue stiffness in contrast to iPSC-PCs, the expression of collagen cross-linkage-associated genes was examined also in relation to native tissue (hRV). The type of collagen crosslinking influences stiffness, collagen degradation, and the reversibility of fibrosis [69]. While for the CM+Fb+EC BCTs containing dermal fibroblasts the expression of fibrosis-related genes such as the enzyme lysyl oxidase (LOX), which covalently cross-links collagens, was found to be not significantly different from the human heart sample (which contained cardiac Fb), we found the LOX to be strongly upregulated in the CM+PC+EC group together with TIMP1, which restricts protein degradation and thereby indirectly promotes ECM deposition. Likewise, other cardiac Fb activity and ECM-associated genes, some of them also commonly used as fibrosis markers [67,70], were almost exclusively upregulated in the CM+PC+EC group compared to CM+Fb+EC (Figure 5B), which supports our observations on increased tissue stiffness and ECM deposition. Brain natriuretic peptide (BNP or NPPB), a prognostic indicator in heart failure, fibrosis, and hypertrophy, showed elevated levels as well. At the same time, genes described as anti-fibrotic or genes which are downregulated during fibrosis [68,71,72] were found to be upregulated, though not significantly, in the CM+Fb+EC group compared to the CM+PC+EC group.

Since there was no external stimulation of fibrosis in our model, we assume that intercellular crosstalk between the different cell types is the key to the underlying mechanism. Our PC-like cells displayed a higher expression of TGFBR1 (Figure 2H), and therefore a presumably higher sensitivity to activating EC-mediated signaling in our multi-cellular model, leading to the more pronounced functional effects in the CM+PC+EC group compared to the tissues generated with fibroblasts.

Further research is mandatory to investigate the modulation of these effects for the development of novel therapeutic strategies; however, factors identified in our model and by others represent potential targets for intervention. A reduction in the LOX activity and collagen cross-linkage either via pirfenidone [28] or by the inhibition of Rho-associated kinases [73] has been investigated in other fibrosis models. Additional options could be the inhibition of myofibroblast differentiation, as has been shown in spinal cord injury, where the inhibition of PC proliferation and its contribution to ECM resulted in facilitated healing and reduced fibrosis [74]. Myofibroblast-secreted POSTN was also identified as an activator of cardiac dedifferentiation in pulmonary hypertension-induced heart failure [75], while the loss of POSTN function could lead to attenuated fibrosis.

## 3. Materials and Methods

### 3.1. Cell Culture

#### 3.1.1. Cultivation of Undifferentiated hPSCs

The hPSC clones iPSC6 (HSC1285_T-iPS2, MHHi006-A) [76], hES3_αMHC-Neo (hESC_αMHC) [77], iPSC9_eGFP (hCBiPSC2_eGFP, based on MHHi009-A), and iPSC9_RedStar (hCBiPSC2_RedStar_Nuc__αMHC-Neo, MHHi009-A-4) [78,79] were cultivated as a feeder-free monolayer culture [80]; for more detail, see the Supplementary Information. All the used hPSC lines retained a normal karyotype, stained positive for pluripotency markers by flow cytometry and immunofluorescence staining, and were regularly tested negative for mycoplasma contamination. Differentiation experiments were started after the monolayer cultures were passaged at least two times.

#### 3.1.2. Differentiation, Selection, and Maintenance of Pericytes

The differentiation of pericytes was performed using a protocol modified from Orlova et al. [15,33]. On day-3 (D-3), iPSC6 or iPSC9_RedStar cells were detached with dispase (1 mg/mL in DMEM/F12; Life Technologies, Paisley, UK) and seeded as colonies on Matrigel-coated (1:100; BD Biosciences, Heidelberg, Germany) 6 well plates in 2 mL mTeSR (StemCell Technologies, Vancouver, BC, Canada). From D0, the cells were cultivated with BMP-4 (30 ng/mL; R&D Systems, Minneapolis, MN, USA), VEGF-A (50 ng/mL; PeproTech, Rocky Hill, NJ, USA) and CHIR99021 (1.5 µM; Institute for Organic Chemistry, Leibniz University Hannover, Hanover, Germany) in the differentiation medium APEL (StemCell Technologies, Vancouver, BC, Canada) for three days. Afterwards, the culture medium was supplemented with VEGF-A (50 ng/mL) and SB431542 (1 µM; Institute for Organic Chemistry, Leibniz University Hannover, Hanover, Germany). On D10, the PDGFRβ^+^/CD31^−^ fraction was sorted via fluorescence activated cell sorting (FACS), cultivated in EGM-2 (Lonza, Basel, Switzerland) for 4 days and further matured in PGM (pericyte growth medium, Promocell, Heidelberg, Germany). PC-related surface marker expression was analyzed by flow cytometry, RT- and qRT-PCR and immunofluorescence staining. Differentiated iPSC-PCs were maintained in PGM, detached using TrypLE Select (Life Technologies, Paisley, UK) at 80–90% confluence, and seeded in a 1 to 4 ratio. Passages between P4 and P7 were used for functional assays and tissue production. Primer sequences, primary, and secondary antibodies used to characterize differentiated PCs are listed in Tables S4–S6, respectively.

#### 3.1.3. Differentiation and Selection of Cardiomyocytes and Endothelial Cells

Human PSC-derived CMs were generated in suspension culture using the protocol described by Dahlmann et al. [51]; for details see Supplementary Information. Differentiation was initiated via bi-phasic modulation of the Wnt signal [81]; the stable integration of a cardiac-specific αMHC promoter-driven neomycin resistance cassette enabled the purification of CMs. The pure cardiomyocyte-containing aggregates, termed cardiac bodies (CB), were dissociated with the STEMdiff Cardiomyocyte Dissociation kit (StemCell Technologies, Vancouver, BC, Canada) for further analysis and tissue formation.

The differentiation of iPSC-derived ECs was performed as described earlier [20,42]; for details see Supplementary Information. The resulting, CD31^+^ iPSC-ECs were used for tissue formation and co-culture assays directly after magnetic cell separation or in P1.

Primary and secondary antibodies used to characterize differentiated CMs and ECs are listed in Tables S5 and S6, respectively.

#### 3.1.4. Preparation and Cultivation of Bioartificial Cardiac Tissues (BCT)

For single-cell cardiomyocyte-based BCTs (SC-BCTs), the CBs were dissociated after selection, and 1 million CMs were used for tissue preparation as described [22]. CMs were mixed with the respective number of other cell types (Figure 3B and Figure 4A) in 100 μL of BCT medium [22] per tissue. An extracellular matrix mixture (150 μL/BCT) composed of 0.9 mg/mL rat collagen type I (Trevigen, Gaithersburg, MD, USA), 10% Matrigel, and 2.5% 0.4 M NaOH was added. The cell-matrix mixture was poured into a custom-made silicon mold containing two titanium rods (distance 6 mm; initial slack length) and solidified at 37 °C for 30 min. Then, the construct was covered by 5 mL of BCT medium with 60 μM of L-ascorbic acid (Sigma-Aldrich, St. Louis, MO, USA) and cultured under standard cell culture conditions with medium change every 1 or 2 days. When BCTs were stimulated with TGFβ1 (5 ng/mL; Peprotech, Rocky Hill, NJ, USA) between D7 and D14, medium was refreshed every day. For all tissues, a growing static stretch (G-stretch) was applied through stepwise elongation by 400 µm on days 7, 11, 15, and 19.

Primer sequences, primary-, and secondary antibodies used to characterize BCTs are listed in Tables S4–S6, respectively.

### 3.2. Force Measurement

Mechanical forces of the tissues were measured on day 21 of tissue cultivation in BCT medium at 37 °C, 5% CO_2_ levels using a custom-made bioreactor system and analysis software (Central Research Workshop, Hannover Medical School, Hanover, Germany) [21] as previously described [22].

In brief, active contraction force and Frank–Starling mechanism was determined at increasing preload (in 100 or 200 μm increments until 1 mm in total) by measuring the response to electric stimulation (5×) with biphasic pulses (10 ms, ±25 V). Passive force for each preload step was defined by the difference between baselines at the actual step and at the original length (L_0_). For the analysis of the force–frequency relationship (FFR) and the frequency-dependent acceleration of relaxation (FDAR), tissues were electrically stimulated at 1, 2, and 3 Hz at maximum preload. For post-rest potentiation (PRP) 60 s of high frequency (≥5 Hz) electrical stimulation was applied, then the first 5 spontaneous peaks were recorded and the values were expressed in percentage of the 1st peak height. Data evaluation was performed via a self-developed MATLAB (MathWorks, Natick, MA, USA) script. BCT diameter was measured from bright field images (microscope SteREO Discovery.V8, Zeiss, Jena, Germany) and cross-sectional areas were calculated assuming a circular geometry. Elastic moduli were calculated from the plots of strain and passive tension [54].

### 3.3. Gene Expression Analysis

Microarray (GEO accession number: GSE145957) and sequencing data (GEO accession number: GSE146150) were generated by the Research Core Unit Genomics (RCUG) at Hannover Medical School. For more technical detail on raw data processing and normalization see Supplementary Information. Non- or very low-expressed genes, as well as gender-specific transcripts were removed from analysis (cut-off was set at base mean >20 A.U. for the Microarray and base mean >10 RPKM for RNAseq). The global analysis of data sets (Tables S1 and S2), including principal component analysis and multiple group comparison were performed with Omics Explorer 3.5 (Qlucore, Lund, Sweden) and Perseus (MPI of Biochemistry, Martinsried, Germany) [82], other analyses were performed with the RCUTASv1.8.3 Excel-tool (provided by the Research Core Unit Genomics, Hannover Medical School, Hanover, Germany). A gene set enrichment analysis (GSEA) was carried out with the GSEA software (version 4.0.1, Broad Institute, Cambridge, MA, USA) [83]. Enriched gene sets with FDR <0.05 were significant, data were expressed as normalized enrichment score (NES).

### 3.4. Statistics

Statistical tests were performed using GraphPad Prism 7.04 (GraphPad Software, San Diego, CA, USA). For the comparison of two means, the Student’s *t*-test was used. To compare multiple experimental groups, a one-way analysis of variance (ANOVA) or two-way ANOVA with Tukey post-test was applied. A *p* value < 0.05 was considered to be statistically significant. Unless otherwise indicated, data on graphs are depicted as MEAN ± SEM.

### 3.5. Ethical Statement

The pluripotent stem cell lines iPSC6, iPSC9_eGFP, and iPSC9_RedStar were generated in house; the line HES-3 (ES Cell International Pte Ltd., Singapore) was imported and genetically modified in house to yield hES_αMHC^Neo^ under approval No. 112 by the Robert Koch Institute to import hESCs according to the German Stem Cell Act. Human placental pericytes were purchased from PromoCell (Heidelberg, Germany), human foreskin fibroblasts were obtained from ATCC (Manassas, VA, USA). Written informed consent for scientific use of human cardiac tissue was obtained from all the patients. Sample acquisition from human material was evaluated and accepted by the ethics committee of Hannover Medical School (vote number 2997–2016, approval date 4 January 2016).

## 4. Conclusions

The differentiated and highly purified PC-like cells derived from human iPSCs demonstrated a typical pericyte-like function and gene expression pattern, while showing signs of a cardiac phenotype. In a fully hPSC-based cardiac tissue model, such iPSC-PCs could functionally replace the commonly used primary dermal fibroblasts, leading to the improved organization of sarcomere structures. More interestingly, the cellular interplay in the 3D environment triggered hallmarks of fibrotic tissue response, such as decreased contractility, increased tissue stiffness, the secretion of BNP, and the upregulation of myofibroblast-associated genes. The observed increase in tissue stiffness became boosted when ECs were included in the tissues to further resemble the composition of the human heart. We experienced the deposition of ECM and increased collagen cross-linkage, typical in myocardial interstitial fibrosis, and we could confirm the involvement of PC-like cells in the fibrotic process, as has been shown already in other organs. Since the pathomechanism of fibrosis is not yet fully understood, this tissue model provides us with a powerful tool to investigate the complex cellular interplay involved in fibrosis-related remodeling in vitro. Notably, with our model including all relevant cell types, the fibrotic process does not depend on external TGFβ stimuli. Moreover, our tri-culture tissues can be used as a screening platform for new anti-fibrotic treatments to address the effects on multiple cardiovascular cell types.

## Supplementary Materials

The following are available online at https://www.mdpi.com/1422-0067/21/23/8947/s1.
