# Supplementary material for: Dual Function of iPSC-Derived Pericyte-Like Cells in Vascularization and Fibrosis-Related Cardiac Tissue Remodeling In Vitro
Source: Int J Mol Sci. 2020 Nov 25;21(23):8947. doi: 10.3390/ijms21238947 (PMC7728071; doi:10.3390/ijms21238947)
Supplement: Supplementary file 1 [file ijms-21-08947-s001.zip › ijms-983939-supplementary.pdf]

Dual Function of iPSC-Derived Pericyte-Like Cells in Vascularization and Fibrosis-Related Cardiac Tissue Remodeling In Vitro

Monika Szepes [1,2], Anna Melchert [1,2], Julia Dahlmann [1,3], Jan Hegermann [3,4], Christopher Werlein [5], Danny Jonigk [2,3,5], Axel Haverich [1,2,3], Ulrich Martin [1,2,3], Ruth Olmer [1,3] and Ina Gruh [1,2*]

[1] Leibniz Research Laboratories for Biotechnology and Artificial Organs (LEBAO), Department of Cardiothoracic, Transplantation and Vascular Surgery, Hannover Medical School, 30625 Hannover, Germany; Szepes.Monika@mh-hannover.de (M.S.); Melchert.Anna@mh-hannover.de (A.M.); Dahlmann.Julia@mh-hannover.de (J.D.); Jonigk.Danny@mh-hannover.de (D.J.); Haverich.Axel@mh-hannover.de (A.H.); Martin.Ulrich@mh-hannover.de (U.M.); Olmer.Ruth@mh-hannover.de (R.O.)

[2] REBIRTH - Research Center for Translational Regenerative Medicine, Hannover Medical School, 30625 Hannover, Germany

[3] Biomedical Research in Endstage and Obstructive Lung Disease Hannover (BREATH), Member of the German Center for Lung Research (DZL), Hannover Medical School, 30625 Hannover, Germany; Hegermann.Jan@mh-hannover.de (J.H.)

[4] Institute of Functional and Applied Anatomy, Research Core Unit Electron Microscopy, Hannover Medical School, 30625 Hannover, Germany; Hegermann.Jan@mh-hannover.de

[5] Institute of Pathology, Hannover Medical School, 30625 Hannover, Germany; Werlein.Christopher@mh-hannover.de (C.W.)

* Correspondence: Gruh.Ina@mh-hannover.de; Tel.: +49-511-532-8901

SUPPLEMENTARY INFORMATION

Cell and tissue culture

All cells, cell lines and tissues were maintained and differentiated under standard cell culture conditions (37˚C, 5% CO_2).

Cultivation of fibroblasts, endothelial cells and pericytes

Human foreskin fibroblasts (hFF; ATCC) were expanded in fibroblast medium (DMEM with 10% FBS, 1% Non-essential Amino acids, 1 mM L-Glutamine, all Life Technologies). Medium was changed every 2-3 days and the cells were passaged in a 1:3 ratio using Trypsin / EDTA Solution (Biochrom) once confluency was reached. Proliferating hFFs were directly used for the experiments or were mitotically inactivated by gamma irradiation.

Human umbilical cord vein endothelial cells (HUVEC) and human placental pericytes (hPC-PL; PromoCell) were maintained in Endothelial Growth Medium-2 (EGM-2; Lonza) and

Pericyte Growth Medium (PGM; PromoCell), respectively. At 80-90% confluency, cells were dissociated using Trypsin / EDTA Solution and re-seeded in a 1:3 ratio. Fresh medium was added every 2-3 days. For the experiments described, HUVEC and hPC-PL passages between 3 and 6 were used.

Cultivation of undifferentiated hPSCs

Human PSCs ($4x10^4$ cells/cm^2) were plated on Geltrex-coated (1:400; Thermo Fisher Scientific, Waltham, US) cell culture flasks (TPP) either in feeder-conditioned medium (CM+; DMEM/F12 + Glutamax with 15% KnockOut serum replacement, 1% non-essential amino acids, 0.1 mM β-mercaptoethanol, all Life Technologies) or mTeSR (StemCell Technologies), both containing 10 µM Y27632 Rho kinase inhibitor (RI; Institute for Organic Chemistry, Leibniz University Hannover). Prior to use, CM+ was conditioned for 24 h on confluent irradiated hFFs and complemented with bFGF (Institute for Technical Chemistry, Leibniz University Hannover) in concentrations tested for each hPSC clone (hESC_αMHC: 10 ng/ml, iPSC6: 50 ng/ml, iPSC9_RedStar: 75 ng/ml). Medium was changed daily, monolayer cultures were dissociated with Accutase (Life Technologies) and re-seeded every third day.

Differentiation of cardiomyocytes

PSCs (hESC_αMHC and iPSC9-RedStar, both carrying an αMHC^{Neo} selection cassette) were aggregated to form embryoid bodies (EB) in non-adhesive agarose/DMEM microwells (agarose: NEEO ultra quality, Roth) containing 4700 pyramid-shaped microcavities prepared by soft lithography as described earlier [1]. For the EB formation (D-3), $5x10^6$ cells were seeded in 3 ml CM+ with 10 µM RI per AgarOwell and aggregated for 24h. On D-2, EBs from 2 AgarOwells (9400 EBs) were pooled in 20 ml CM+ and transferred to an orbital shaker (55 rpm). Differentiation was started with 24h activation of the Wnt pathway using 8 µM CHIR99021, followed by 24 h inhibition of Wnt-pathway using 5 µM IWP-2 (both molecules:

Institute for Organic Chemistry, Leibniz University Hannover), in 20 ml RB- medium (RPMI1640 with 2% B-27 Supplement minus insulin, both Life Technologies). Between D2 and D7 the cells were cultivated in RB- medium without any supplements and fresh medium was added daily. On D7, the medium was changed to basic serum free medium (bSF) composed of DMEM supplemented with 1% non-essential amino acids, 1 mM L-glutamine, 5.6 mg/l transferrin (Sigma-Aldrich), 37.2 µg/l sodium-selenite (Sigma-Aldrich), and 10 µg/ml insulin (SAFC Biosciences). Purification of cardiomyocytes was performed by antibiotic selection in bSF medium supplemented with 200 µg/ml G418 (Sigma-Aldrich) from D10 to D17 and the efficiency of the selection was analysed via flow cytometry by detecting the percentage of cardiac troponin T (cTnT), α-sarcomeric actinin (αSA), and myosin heavy chain (MYH) positive cells. On D18, the medium was changed to bioartificial cardiac tissue (BCT) medium containing DMEM with 12% horse serum (Life Technologies), 1 mM L-glutamine, 1% penicillin/ streptomycin (10 U/ml penicillin, 10 µg/ml streptomycin, Biochrom GmbH), and 10 µg/ml insulin.

Differentiation of endothelial cells

To induce endothelial cell differentiation [2], single cells of iPSC9_eGFP were seeded in agitated Erlenmeyer flasks containing mTeSR + 10 µM RI (D-1). Aggregate formation was allowed for 24 hours on an orbital shaker at 55 rpm. On D0 of the differentiation, medium was replaced by N2B27 medium (1:1 mixture of DMEM/F12 and Neurobasal media with N2 and B27; all Thermo Fischer) supplemented with BMP4 (25 ng/ml) and CHIR90221 (7.5 µM) for 2 days without medium exchange. From D3 to D7, cultures were maintained in StemPro-34 medium (Thermo Fisher) supplemented with VEGF-A (260 ng/ml, Peprotech) and 2 µM Forskolin (Sigma-Aldrich) with daily medium exchanges. Cultures were dissociated on D7 of differentiation using Collagenase II (Worthington). The $CD31^+$ endothelial cell population was purified by magnetic cell separation (MACS). The resulting iPSC-ECs were maintained on

fibronectin-coated (25 µg/ml, Corning) plates in EGM-2 medium and passaged 1:3 with Accutase at 80% confluency.

In vitro EC-PC co-culture model

Co-cultivation of iPSC-PCs and iPSC- ECs in 3-D fibrin matrices was performed as described by Rohringer *et al.* [3] for HUVECs and adipose-derived stem cells. Round coverslips were placed into a 12-well plate and a 25 µl droplet of the fibrinogen component (2.5 mg/ml final concentration) of TISSEEL (Baxter) was pipetted on top. Meanwhile, $1x10^5$ iPSC-ECs alone or in combination with 3x105 human foreskin fibroblasts (hFF) or $3x10^5$ iPSC-PCs were resuspended in 25 µl of the thrombin solution (0.2 U/ml with 16 nmol $CaCl_2$), then mixed with the fibrinogen droplet. Gels were polymerised for 30 min at 37˚C and either EGM-2 or BCT medium was added. The matrices were cultivated for 7 days with medium change on day 2 and 5.

Human tissue acquisition, sampling and preparation

Human tissue samples were acquired from explanted hearts or tissue from cardiac-assist device implantation as part of Clinical Research Group (KFO) 311 program. Fresh human samples were processed immediately after explantation of the organ / heart tissue in a standardized and localized fashion. Every specimen was macroscopically and histologically examined by pathologists regarding safety issues (i.e. infections) and viability of the provided material.

Microscopy and flow cytometry

Microscopic analysis of living or fixed cells and tissues

For assessment of cellular morphology in co-culture models and during BCT formation, live cell imaging was performed using an AxioObserver Z1 fluorescence microscope (Zeiss) with a 37˚C humidity chamber and 5% CO_2 level. Distribution of CMs was visualized by 25

nM tetramethylrhodamine (TMRM, Thermo Fisher) [4]. To acquire overview and in-depth images the AxioVision software modules MosaiX and Z-Stack were used. Evaluation of GFP^+ endothelial networks during cultivation was done by ImageJ.

For immunofluorescence staining, adherent cells were fixed with 2% paraformaldehyde (PFA, Sigma-Aldrich) for 10 min and glass slide-mounted BCT sections, previously embedded in Tissue-Tek O.C.T Compound (Sakura Finetek Europe), were fixed with 4% PFA for 4 minutes. Samples were blocked and permeabilized using Tris-buffered saline containing 0.25% Triton-X 100 and 5% donkey serum. Incubation with primary antibody was performed either for an hour at room temperature or overnight at 4 ˚C. Secondary antibodies were added for 30 min at room temperature. Both primary and secondary antibodies were diluted in phosphate buffered saline (w/o Ca^{2+} and Mg^{2+}) + 1% bovine serum albumin (BSA) and used in ratios listed in **Table S.5** and **Table S.6**. Nuclei were stained for 15 min with 4',6-diamidino-2-phenylindole (DAPI). Images were taken using an AxioObserver A1 fluorescence microscope and analyzed by AxioVision software 4.71 (both Zeiss). Directionality analysis of BCTs was performed by ImageJ using the Fourier spectrum analysis based ‘Directionality’ plug-in (created by Jean-Yves Tinevez) as described [5].

Co-cultures in 3-D fibrin matrices were fixed with 4% PFA for 2 hours. Coverslips were carefully removed from the 12-well plates, stained as described above and mounted on top of a glass slide. Z-stack images were taken with the AxioObserver Z1 microscope and processed by ImageJ.

Histological stainings

For Masson-Goldner staining (Roth), 7 µm thick slices of paraffin-embedded BCTs were used. Following deparafinization, the slides were stained and mounted according to manufacturer´s instructions. The collagen-specific Sirius red F3B (Sigma) staining was

performed as described earlier [6]. Stained sections were analysed using an Olympus microscope (BX40) with a color camera (Axiocam 512, Zeiss).

Visualisation of ECM deposition was carried out using Gömöri's Trichrome staining. Representative images were taken using an Olympus BX43 light microscope equipped with an Olympus CS50 camera using Olympus cellSens Software. For quantification of ECM deposition, individual thresholds for fibres marked in red and blue were applied to RGB images using ImageJ software [7] and the resulting tissue parts where quantified, as previously described [8].

Flow cytometry

Live cell staining with directly labelled antibodies against surface markers (all Miltenyi Biotec) was performed according to manufacturer´s instructions. Prior to antibody staining for intracellular markers, cells were fixed with ice-cold 90% methanol for 15 min. Primary antibodies or the respective isotype controls, diluted in a PBS w/o Ca^{2+} and Mg^{2+} (Life Technologies) buffer containing 0.1% Triton-X 100 (Sigma-Aldrich) and 0.5 % BSA (Sigma-Aldrich), were incubated with the cells for 1 hour. This was followed by a 30 min incubation period with the appropriate secondary antibody.

All measurements were performed with the Accuri C6 (BD Biosciences) flow cytometer and data was analysed using FlowJo_V10. Primary antibodies and their dilutions are listed in **Table S.5**, secondary antibodies are listed in **Table S.6**. Fluorescence activated cell sorting (FACS) was carried out by the Research Facility Cell Sorting of MHH using a MoFlo XDP high-speed cell sorter (Beckman-Coulter).

Electron microscopy

BCTs were placed into cardioplegic solution (St. Thomas' Hospital solution I, Dr. Franz Köhler Chemie GmbH) for 5 min to relax the sarcomeres, then fixed in 0.15 M HEPES buffer

with 1.5% formaldehyde and 1.5% glutaraldehyde and stored at 4°C until embedding. Embedding and image acquisition was carried out by the Research Core Unit Electron Microscopy at Hannover Medical School as described by Kensah *et al.* [4].

Gene expression analysis

Analysis of RNA expression by qRT-PCR

Total RNA was isolated with the RNeasy Kit (Macherey-Nagel) from primary cells and hPSC-derivatives. From tissues and BCTs RNA isolation was done using acid guanidinium thiocyanate-phenol-chloroform extraction (TRIzol, Invitrogen) followed by DNAse (Thermo Fisher) treatment. In both cases 500 ng RNA per sample was used for random primed cDNA synthesis with the RevertAid H Minus First strand cDNA Synthesis kit (Thermo Fisher) according to manufacturer’s instructions. Contamination with genomic DNA was excluded by using a cDNA synthesis control without reverse transcriptase.

Quantitative real-time PCR was performed in triplicates using the Absolute qPCR SYBR Green Mix (Thermo Fisher) and a Mastercycler ep realplex^2 PCR cycler (Eppendorf). All primer pairs for qRT-PCR were used at 60˚C annealing temperature. The relative expression ratio between the target gene and reference gene transcript levels was calculated as described by Pfaffl [9]. The target gene-dependent controls for primer pairs are indicated in the Results section. Primer pairs used were obtained from Eurofins Genomics or SeqLab and are detailed in **Table S.4**.

Microarray analysis

Microarray design, measurement, data pre-processing and normalization was performed by the Research Core Unit Genomics of Hannover Medical School. The microarray utilized represents a refined version of the Whole Human Genome Oligo Microarray 4x44K v2 (Design ID 026652, Agilent Technologies), called ‘026652QM_RCUG_HomoSapiens’ (Design ID

084555) created with Agilent's eArray portal using a 1x1M design format for mRNA expression as template. All non-control probes of design ID 026652 have been printed five times within a region comprising a total of 181560 features (170 columns x 1068 rows). Four of such regions were placed within one 1M region giving rise to four microarray fields per slide to be hybridized individually (Customer Specified Feature Layout). Control probes required for proper Feature Extraction software operation were determined and placed automatically by eArray using recommended default settings.

375 ng of total RNA were used for synthesis of aminoallyl-UTP-modified (aaUTP) cRNA with the Quick Amp Labeling kit, no dye (Agilent Technologies) according to the manufacturer's recommendations, except that reaction volumes were quartered and contained NTP-mix was exchanged by NTP Set (ATP, CTP, GTP, UTP) and aminoallyl-UTP (Fermentas, Thermo Fisher Scientific). Final NTP concentrations used for in-vitro transcription were 2.5mM (ATP, CTP, GTP), 1.88 mM UTP, and 0.62 mM aaUTP. The labeling of aaUTP-cRNA was performed by use of Alexa Fluor 555 Reactive Dye as described in the Amino Allyl MessageAmp II Kit Manual (both Life Technologies) except that reaction volumes were quartered. Prior to the reverse transcription reaction, 0.75 µl of a 1:1000 dilution of One-Color spike-in Kit stock solution (Agilent Technologies) were added to each 375 ng of total RNA sample.

Slides were scanned on the Agilent Micro Array Scanner G2565CA (pixel resolution 3 µm, bit depth 20). Data extraction was performed with the Feature Extraction Software (V10.7.3.1).

Measurements of on-chip replicates (quintuplicates) were averaged using the geometric mean of processed intensity values of the green channel, gProcessedSignal (gPS) to retrieve one resulting value per unique non-control probe. Single features were excluded from averaging, if they i) were manually flagged, ii) were identified as Outliers by the Feature Extraction Software, iii) lay outside the interval of '1.42 x interquartile range' regarding the

normalized gPS distribution of the respective on-chip replicate population, or, iv) showed a coefficient of variation of pixel intensities per Feature that exceeded 0.5.

Averaged gPS values were normalized by quantile normalization followed by global linear scaling. For the latter approach, all quantile normalized (QN) gPS values of one sample were multiplied by an array-specific scaling factor. This factor was calculated by dividing a ‘reference 75th Percentile value’ (set as 1500 for the whole series) by the 75th Percentile value of the particular microarray to be normalized ($Array_i$). Accordingly, finally normalized (FN) gPS values for all samples (microarray data sets) were calculated by **Equation 1**.

$$FN\ gPS_{Array_i} = QN\ gPS_{Array_i} \times \frac{1500}{75^{th}\ Percentile_{Array_i}} \qquad (1)$$

Finally, a lower intensity threshold (surrogate value) was defined based on intensity distribution of negative control features. This value was fixed at 10 finally normalized gPS units. Those measurements that fell below this intensity cutoff were substituted by the respective surrogate value of 10.

RNA – sequencing (Library generation, sequencing, and raw data processing)

Experimental design, sequencing, data pre-processing and normalization was performed by the Research Core Unit Genomics of Hannover Medical School. 200ng of total RNA per sample were utilized as input for mRNA enrichment procedure with Poly(A) mRNA Magnetic Isolation Module (NEBNext®, New England Biolabs) followed by stranded cDNA library generation using Ultra II Directional RNA Library Prep Kit for Illumina (NEBNext®). All steps were performed as recommended in user manual except that all reactions were downscaled to 2/3 of initial volumes. Furthermore, one additional purification step was introduced at the end of the standard procedure, using 1x Agencourt® AMPure® XP Beads (Beckman Coulter). The cDNA libraries were barcoded by dual indexing approach, using Multiplex Oligos for Illumina – 96 Unique Dual Index Primer Pairs (NEBNext®) and all libraries were amplified with 7

cycles of final PCR. Quantification of libraries was performed by use of the Qubit® dsDNA HS Assay Kit (ThermoFisher Scientific) and the fragment length distribution of individual libraries was monitored using Bioanalyzer High Sensitivity DNA Assay (Agilent Technologies).

Equal molar amounts of twelve individually barcoded libraries were pooled. Accordingly, each analyzed library constitutes 8.3% of overall flowcell capacity. The library pool was denatured with NaOH and was finally diluted to 1.8 pM according to the Denature and Dilute Libraries Guide (Illumina). 1.3 ml of denatured pool was loaded on an Illumina NextSeq 550 sequencer using a High Output Flowcell for single reads.

Raw data processing was conducted by use of the nfcore/rnaseq (version 1.3) bioinformatics best-practice analysis pipeline used for RNA sequencing data at the National Genomics Infrastructure (SciLifeLab Stockholm). The pipeline uses Nextflow, a bioinformatics workflow tool. It pre-processes raw data from FastQ inputs, aligns the reads and performs extensive quality-control on the results. The genome reference and annotation data were taken from GENCODE.org (Homo sapiens; GRCh38; release 29). Normalization and differential expression analysis was performed with DESeq2 (Galaxy Tool Version 2.11.40.2) with default settings.

Gene expression analysis

Table S.1. Description of samples used in microarray analysis (GSE145957).

Sample	*Passage*	*Clone*
hFF (Lot#59733309)	19	SCRC-1041
hFF (Lot#63229645)	20	SCRC-1041
hPC-PL (Lot#3051403.2)	3	Primary
hPC-PL (Lot#4021804.1)	3	Primary
iPSC-EC differentiation 1	1	iPSC9-eGFP
iPSC-EC differentiation 2	1	iPSC9-eGFP
iPSC-EC differentiation 3	1	iPSC9-eGFP
iPSC-PC differentiation 1	4	iPSC6
iPSC-PC differentiation 2	4	iPSC6
iPSC-PC differentiation 3	4	iPSC6
Undifferentiated monolayer	47+7	iPSC6
Undifferentiated monolayer	76+4	iPSC9-eGFP
Undifferentiated monolayer	38+11	hESC_αMHC^{Neo}

Table S.2. Description of samples used in RNA sequencing (GSE146150).

Sample	*Source*	*Age*
Right ventricle 22350/18	Right ventricle	adult
Right ventricle 13705/18	Right ventricle	adult
Right ventricle 16183/18	Right ventricle	adult
hESC-CM 1	CMs (hESC_αMHC^{Neo})	Differentiation D21
hESC-CM 2	CMs (hESC_αMHC^{Neo})	Differentiation D18
hESC-CM 3	CMs (hESC_αMHC^{Neo})	Differentiation D18
hESC-CM BCT with hFF+EC 1	hESC-CMs + iPSC-ECs (iPSC9-eGFP) + hFFs (ATCC-SCRC-1041)	Tissue cultivation D21
hESC-CM BCT with hFF+EC 2	hESC-CMs + iPSC-ECs (iPSC9-eGFP) + hFFs (ATCC-SCRC-1041)	Tissue cultivation D21
hESC-CM BCT with hFF+EC 3	hESC-CMs + iPSC-ECs (iPSC9-eGFP) + hFFs (ATCC-SCRC-1041)	Tissue cultivation D21
hESC-CM BCT with PC+EC 1	hESC-CMs + iPSC-ECs (iPSC9-eGFP) + iPSC-PCs (iPSC6)	Tissue cultivation D21
hESC-CM BCT with PC+EC 2	hESC-CMs + iPSC-ECs (iPSC9-eGFP) + iPSC-PCs (iPSC6)	Tissue cultivation D21
hESC-CM BCT with PC+EC 3	hESC-CMs + iPSC-ECs (iPSC9-eGFP) + iPSC-PCs (iPSC6)	Tissue cultivation D21

Table S.3. – Genes marked on Figure 5.B. with values

Significant	-Log(p)	Difference	Gene name	Color	CM+hFF+EC1	CM+hFF+EC2	CM+hFF+EC3	CM+PC+EC1	CM+PC+EC2	CM+PC+EC 3
+	2.8182	5.3332	CAV3		4.5850	4.7004	6.7142	0.0000	0.0000	0.0000
+	2.1648	1.1802	HEY2		8.1548	7.8580	7.9129	6.3750	6.9658	7.0444
+	2.5675	2.6121	KCND3		8.3174	7.7004	8.5353	5.0000	6.0444	5.6724
+	2.8639	0.9939	GJC1		10.8681	10.6119	10.7474	9.5488	9.8765	9.8202
+	2.9261	2.4608	NR2F2		8.3219	7.9366	7.4998	5.4919	5.7549	5.1293
+	4.1742	-2.1650	RYR3		3.3219	3.0000	3.1699	5.1699	5.3576	5.4594
+	2.5310	-4.9437	SLN		4.0875	6.3923	4.9069	10.3083	10.5430	9.3663
+	2.5310	-4.9437	SLN		4.0875	6.3923	4.9069	10.3083	10.5430	9.3663
+	2.2437	0.9279	MYLK3		13.2063	13.0308	13.1599	12.1280	11.9669	12.5184
+	4.0548	-4.3663	KCND2		2.0000	1.5850	1.5850	6.5546	5.8074	5.9069
	0.9219	0.9083	MYH6		17.8956	17.9944	18.4205	16.3385	17.5376	17.7095
	0.7244	0.7886	MYH7		15.5903	14.8273	14.5671	13.4306	14.6975	14.4908
	1.0861	2.1257	CACNA1G		10.3630	10.7582	10.7740	6.6865	9.4676	9.3641
	0.9820	1.4976	MYOM2		12.0918	11.9766	12.4991	9.3264	11.6184	11.1299
	1.1014	0.9179	RYR2		14.2981	14.1226	14.1926	12.5191	13.5639	13.7764
	1.2257	0.9285	MYL2		14.6844	14.4161	14.2195	12.8531	13.8564	13.8251
	0.4834	0.3759	CKMT2		12.6983	12.9281	13.0327	11.8823	12.9571	12.6922
	1.6152	1.6447	MYOM3		9.9425	9.8595	9.9929	7.4594	9.0661	8.3354
	1.8989	1.3585	CASQ2		13.4274	13.0544	13.6330	11.4843	12.3351	12.2201
	1.1099	0.8831	MYOM1		13.9247	13.8640	14.1734	12.3807	13.4431	13.4890
	1.0710	0.4990	TNNT2		16.5221	16.6439	16.8080	15.7639	16.2779	16.4353
	1.6029	1.2742	TTN		17.1360	16.6282	16.2702	15.1223	15.1582	15.9314
	0.7734	0.5071	KCNH2		12.2589	12.3219	12.5567	11.3015	12.2270	12.0878
	1.5235	0.8496	MYL7		16.0645	15.9068	16.3040	14.8808	15.1758	15.6699
	1.2807	0.5295	ACTC1		17.5127	17.4765	17.2358	16.5978	16.8437	17.1951
	0.8903	0.5365	CACNA1C		12.5918	12.6475	12.4788	11.5338	12.0875	12.4873
	0.4723	-0.4611	KCNA5		8.2761	8.4051	8.8948	8.2288	9.3597	9.3707
	1.4881	1.2666	CAVIN4		11.2732	11.0861	10.4409	9.1997	9.5661	10.2348
	0.6370	1.0513	RYR1		5.5236	4.8580	6.5078	3.4594	4.9542	5.3219
	0.3787	0.3964	CKM		13.8894	13.8288	14.2268	12.7429	13.9715	14.0416
+	2.6841	-1.1572	LOXL2		12.1984	12.1360	11.6944	13.2321	13.1624	13.1059
+	1.9587	-3.3018	VTN		5.5850	5.2479	3.9069	9.2668	7.8009	7.5774
+	1.9057	-1.8749	ANG		3.1699	4.1699	3.0000	5.4919	5.6147	4.8580
+	4.9960	-3.3589	SERPINF2		0.0000	0.0000	0.0000	3.1699	3.3219	3.5850
+	3.2887	-4.8668	TCF21		0.0000	0.0000	1.0000	5.5850	4.5236	5.4919
+	2.1700	-1.7528	COL5A1		13.4952	13.2694	12.7095	15.3072	14.4547	14.9705
+	2.6759	-2.9052	POSTN		9.7398	9.1573	9.6129	13.0619	12.3856	11.7781
+	1.7161	-3.4617	IL1A		4.1699	5.0000	3.7004	9.3576	6.5850	7.3129

Significant	-Log(p)	Difference	Gene name	Color	CM+hFF+EC1	CM+hFF+EC2	CM+hFF+EC3	CM+PC+EC1	CM+PC+EC2	CM+PC+EC 3
+	2.2657	-1.0189	LOXL3		7.9944	8.4346	8.0768	8.9658	9.4094	9.1874
+	1.8884	-3.4343	DCN		10.0954	9.0634	9.9366	14.4879	11.9524	12.9582
+	3.1697	-3.3488	LOX		9.8074	9.7731	9.4939	13.7119	12.6555	12.7532
+	2.0106	-3.9515	COL6A6		3.4594	2.3219	3.4594	8.4878	5.9069	6.7004
+	2.1903	-2.5502	CFH		7.6073	6.4757	7.0980	10.1787	9.7194	8.9337
+	2.2650	-2.0080	COL1A2		15.1894	14.4261	14.7788	17.2866	16.2741	16.8577
+	2.0757	-1.8239	THBS4		9.8626	10.6046	9.9024	11.3701	12.1883	12.2828
+	2.1812	-3.1803	SERPINE 2		9.2550	8.7781	9.3015	13.2509	11.2161	12.4086
+	2.3064	-2.5328	COL3A1		15.5908	15.0890	15.0274	18.5069	17.0743	17.7244
+	1.6935	-4.6333	IL6		2.5850	4.2479	2.3219	9.8009	6.2095	7.0444
+	2.8675	-2.6805	DLK1		9.3061	9.8872	10.0307	12.8840	12.0070	12.3745
+	2.2603	-2.9649	COL1A1		14.9970	14.0159	14.0975	18.0839	16.5489	17.3722
+	2.5145	-1.9599	HAS2-AS1		3.7004	4.3219	3.9069	6.3398	5.9773	5.4919
+	2.3473	-2.2549	ITGA11		9.5661	9.5118	9.1033	12.3534	11.1459	11.4466
+	2.1819	-1.4733	THY1		10.4200	10.0728	9.5999	11.7237	11.5854	11.2033
+	2.1321	-2.0470	COL14A1		8.1997	7.8265	8.1085	10.5555	9.3129	10.4073
+	1.9673	5.5768	MMP3		7.3576	7.8202	6.4594	3.9069	0.0000	1.0000
+	2.4160	-2.3774	FN1		13.5887	13.7870	13.1157	16.5497	15.4528	15.6211
+	2.7193	-1.4130	VWA1		9.4533	9.6439	9.0028	10.7125	10.7707	10.8556
+	2.0431	-1.8965	SPARC		14.3353	13.8935	13.7226	16.5781	15.4064	15.6563
+	2.2132	-3.5809	LUM		11.1880	10.3387	10.7632	15.5722	13.4803	13.9801
+	1.9247	-2.8694	TIMP1		11.2222	10.9293	10.9549	15.1936	13.1309	13.3902
+	3.1747	-0.9203	P4HB		13.4670	13.5476	13.3810	14.5416	14.2566	14.3584
+	2.3148	-2.5099	CFI		6.0224	6.3923	5.4919	9.1923	8.1997	8.0444
+	2.3755	-3.5900	TGFBI		10.0014	8.9944	9.8781	14.2285	12.4836	12.9318
	0.0227	-0.0154	TGFB2		10.1137	10.1997	10.5565	10.2408	10.0279	10.6475
	0.7070	0.2884	TGFBR2		11.1630	11.2697	10.8681	11.0961	10.6573	10.6821
	1.1827	-0.3914	TGFBR3		9.2527	9.3707	9.0634	9.3663	9.7330	9.7616
	0.9316	-2.2570	IGF1		5.0444	6.0224	3.0000	8.3443	6.1898	6.3038
	1.0482	-0.7894	TNC		12.1808	11.2779	11.2491	12.6939	12.0888	12.2932
	1.0823	-0.6658	TGFBR1		10.2204	10.0741	10.0430	11.3332	10.3955	10.6064
	0.2479	-0.3181	DDR2		10.0168	9.4574	9.1497	10.7288	9.3083	9.5411
	2.2230	-0.5979	TIMP2		12.0217	11.8952	11.9248	12.7330	12.3677	12.5348
	0.9081	2.3900	DPT		5.0000	3.1699	4.3923	3.8074	0.0000	1.5850
	1.6599	-0.6252	VIM		14.2067	14.0846	14.1625	15.0983	14.6982	14.5330
	0.0931	0.3579	CCN2		12.1926	12.3046	10.6348	11.1567	9.2715	13.6300
	0.2342	-0.5011	NGFR		6.3398	8.2143	5.8826	6.9773	8.1948	6.7682
	1.6615	-2.9969	NPPB		11.0063	11.6913	9.2691	13.6808	14.3198	12.9567
	0.4669	-0.7283	NT5E		8.0168	7.6221	7.8329	9.8811	7.8329	7.9425

Significant	-Log(p)	Difference	Gene name	Color	CM+hFF+EC1	CM+hFF+EC2	CM+hFF+EC3	CM+PC+EC1	CM+PC+EC2	CM+PC+EC 3
	0.6117	-1.4651	CCL2		7.7074	8.7279	6.3750	10.5546	7.6795	8.9715
	1.6906	-0.2598	MIF		9.5622	9.6653	9.6653	9.8626	9.8025	10.0070
	0.7557	-0.4348	TGFB3		7.8455	7.9484	8.0715	8.8948	8.0607	8.2143
	0.3061	-1.5798	TNF		1.0000	4.2479	0.0000	5.1293	0.0000	4.8580
	1.0311	-2.1541	IL1B		7.4179	7.3219	6.6865	11.1818	8.1033	8.6036
	1.5046	-2.1726	COMP		2.0000	2.3219	3.4594	5.4919	3.8074	5.0000
	1.1261	-1.5682	SPP1		6.8580	7.8009	7.6724	9.7515	9.4263	7.8580
+	2.9651	-1.2199	NRP2		9.9744	9.9672	10.2095	11.3354	11.0362	11.4393
	0.3559	1.1944	VWF		6.2288	7.2384	4.5850	5.3923	6.4919	2.5850
	0.0743	0.1295	TEK		6.9887	6.8074	5.8074	7.3576	6.1293	5.7279
	0.1749	0.1519	KDR		11.3348	11.5073	10.5362	11.1630	11.0539	10.7056
	1.3211	-0.3824	NRP1		13.1597	12.9090	12.7981	13.1726	13.4049	13.4366
	0.6362	1.0598	CDH5		9.8057	10.5689	8.7415	8.4009	9.6653	7.8704
	0.6585	0.8710	PECAM1		10.6883	11.6457	10.5353	9.7814	11.0403	9.4346
	0.7082	1.2913	CD34		10.9432	11.1892	9.2360	8.9915	10.2131	8.2900
	0.9517	1.0431	VEGFD		2.8074	2.8074	3.9069	2.8074	2.0000	1.5850
	1.0792	0.4999	SMAD6		9.3531	9.3837	9.9929	9.1849	8.9658	9.0795
	0.6718	2.2997	MMP9		6.6582	8.4717	4.9542	4.0000	6.5999	2.5850
	0.7592	0.9175	CORIN		12.0203	11.8956	12.6488	10.2819	11.5906	11.9396

Oligonucleotides

Table S.4. Primer sequences for qRT-PCR.

Primer	*Accession*	*Sequence*	*Efficiency*	*Product Size*
**β-actin fwd**	NM_001101	GAGCACAGAGCCTCGCCTTT	1.91	212 bp
**β-actin rev**		ATCCTTCTGACCCATGCCCA		
**CD31 fwd**	NM_000442	AGACGTGCAGTACACGGAAG	1.97	120 bp
**CD31 rev**		CTTTCCACGGCATCAGGGA		
**CD144 fwd**	NM_001795	ATCAAGCCCATGAAGCCTCT	1.98	261 bp
**CD144 rev**		AATGCTATGCCTAGCCGCAT		
**COL1A1 s**	NM_000088.3	CTCGAGGATTGCCCGGAAC	1.86	231 bp
**COL1A1 ras**		CAGTAGCACCATCATTTCCACG		
**COL3A1 s**	NM_000090.3	GGAAGCCAGAACCATGCCAA	2.00	137 bp
**COL3A1 ras**		CTGTGGGCAAACTGCACAAC		
**GAPDH s**	NM_002046.7	GGAGCGAGATCCCTCCAAAAT	1.91	104 bp
**GAPDH ras**		GCAAATGAGCCCCAGCCTTC		
**KDR fwd**	NM_002253	GGAGCCTACAAGTGCTTC	2.00	221 bp
**KDR rev**		GGAACAAATCTCTTTTCTGG		
**NANOG fwd**	NM_024865	GGACACTGGCTGAATCCT TCC	1.87	143 bp
**NANOG rev**		CTCGCTGATTAGGCTCCAACC		
**NG2 fwd**	NM_001897	GCTTTGACCCTGACTATGTTGGC	1.87	195 bp
**NG2 rev**		TCCAGAGTAGAGCTGCAGCA		
**OCT4 fwd**	NM_002701	TCCCATGCATTCAAACTGAGG	1.99	104 bp
**OCT4 rev**		CCTTTGTGTTCCCAATTCCTTCC		
**PDGFRα fwd**	NM_006206	TGCCCGAGGAATGGAGTTTT	1.96	176 bp
**PDGFRα rev**		ACTTCACGGGCAGAAAGGTACTG		
**PDGFRβ fwd**	NM_002609	CAGTAAGGAGGACTTCCTGGAG	2.01	178 bp
**PDGFRβ rev**		CCTGAGAGATCTGTGGTTCCAG		
**T-Bra fwd**	NM_003181	CGGAACAATTCTCCAACCTATT	2.07	357 bp
**T-Bra rev**		GTACTGGCTGTCCACGATGTCT		

Antibodies

Table S.5. Primary antibodies.

Antibody	*Isotype*	*Company*	*Dilution*
Calponin 1 #sc-58707	mouse IgG1	Santa Cruz Biotechnology	1:50
CD105-APC #130-099-125	mouse IgG1	Miltenyi Biotec GmbH	1:11
CD140b-APC #130-105-280	human IgG1κ	Miltenyi Biotec GmbH	1:11
CD140b-PE #130-105-321	human IgG1κ	Miltenyi Biotec GmbH	1:11
CD144-PE #130-100-714	human IgG1κ	Miltenyi Biotec GmbH	1:11
CD146-APC #130-097-942	mouse IgG1	Miltenyi Biotec GmbH	1:11
CD31-APC #130-092-652	mouse IgG1	Miltenyi Biotec GmbH	1:11
CD73-APC #130-097-945	mouse IgG1κ	Miltenyi Biotec GmbH	1:11
CD90-APC #130-097-935	mouse IgG1	Miltenyi Biotec GmbH	1:11
KDR-PE #130-093-598	mouse IgG1κ	Miltenyi Biotec GmbH	1:11
MYH (MF20) #AB_2147781	mouse IgG2b	Hybridoma Bank	1:50
NG2 #sc-80003	mouse IgG2a	Santa Cruz Biotechnology	1:50
Sarcomeric α-actinin #A7811	mouse IgG1	Sigma-Aldrich	1:800
Troponin-T #MS-295-P	mouse IgG1	Richard Allan Scientific	1:100
Vimentin #ab92547	rabbit IgG	Abcam	1:500
αSMA #DM001	mouse IgG2a	Acris Antibodies GmbH	1:50
MLC2a #311011	mouse IgG2b	Synaptic Systems	1:100
MLC2v #ab79935	rabbit IgG	Abcam	1:100
CD144 #ab33168	rabbit IgG	Abcam	1:100
CD31#ab28364	rabbit IgG	Abcam	1:150
vWF #A0082	rabbit IgG	Dako	1:500

Isotype controls			
Negative Control	mouse IgG1	Dako, Glostrup, DK	
Negative Control	mouse IgG2a	Dako, Glostrup, DK	
Negative Control	mouse IgG2b	Dako, Glostrup, DK	
Mouse IgG1-PE	mouse IgG1	Miltenyi Biotec GmbH	
Mouse IgG1-APC	mouse IgG1	Miltenyi Biotec GmbH	
Rabbit polyclonal IgG	rabbit IgG	Abcam, Cambridge, UK	
REA control (S)-APC	human IgG1κ	Miltenyi Biotec GmbH	
REA control (S)-PE	human IgG1κ	Miltenyi Biotec GmbH	

Isotype controls were used in the same concentration as the respective primary antibody for each staining. REA –Recombinant Engineered Antibody processed in human HEK293 cells

Table S.6. Secondary antibodies.

Species	*Type*	*Fluorescent label*
**Donkey**	Anti-mouse IgG	Alexa Fluor 488
**Donkey**	Anti-mouse IgG	Cy3
**Donkey**	Anti-mouse IgG	Alexa Fluor 647
**Donkey**	Anti-rabbit IgG	Alexa Fluor 647
**Donkey**	Anti-rabbit IgG	Cy3

All secondary antibodies were purchased from Dianova and used in a 1:300 dilution.

References

1. Dahlmann, J.;Kensah, G.;Kempf, H.;Skvorc, D.;Gawol, A.;Elliott, D.A., *et al.* The use of agarose microwells for scalable embryoid body formation and cardiac differentiation of human and murine pluripotent stem cells. *Biomaterials*. **2013**;*34*(10):2463-71. doi:10.1016/j.biomaterials.2012.12.024.
2. Olmer, R.;Engels, L.;Usman, A.;Menke, S.;Malik, M.N.H.;Pessler, F., *et al.* Differentiation of Human Pluripotent Stem Cells into Functional Endothelial Cells in Scalable Suspension Culture. *Stem Cell Reports*. **2018**;*10*(5):1657-72. doi:10.1016/j.stemcr.2018.03.017.
3. Rohringer, S.;Hofbauer, P.;Schneider, K.H.;Husa, A.M.;Feichtinger, G.;Peterbauer-Scherb, A., *et al.* Mechanisms of vasculogenesis in 3D fibrin matrices mediated by the interaction of adipose-derived stem cells and endothelial cells. *Angiogenesis*. **2014**;*17*(4):921-33. doi:10.1007/s10456-014-9439-0.
4. Kensah, G.;Roa Lara, A.;Dahlmann, J.;Zweigerdt, R.;Schwanke, K.;Hegermann, J., *et al.* Murine and human pluripotent stem cell-derived cardiac bodies form contractile myocardial tissue in vitro. *European heart journal*. **2013**;*34*(15):1134-46. doi:10.1093/eurheartj/ehs349.
5. Li, J.;Minami, I.;Shiozaki, M.;Yu, L.;Yajima, S.;Miyagawa, S., *et al.* Human Pluripotent Stem Cell-Derived Cardiac Tissue-like Constructs for Repairing the Infarcted Myocardium. *Stem Cell Reports*. **2017**;*9*(5):1546-59. doi:10.1016/j.stemcr.2017.09.007.
6. Yap, L.;Wang, J.W.;Moreno-Moral, A.;Chong, L.Y.;Sun, Y.;Harmston, N., *et al.* In Vivo Generation of Post-infarct Human Cardiac Muscle by Laminin-Promoted Cardiovascular Progenitors. *Cell Rep*. **2019**;*26*(12):3231-45 e9. doi:10.1016/j.celrep.2019.02.083.
7. Schindelin, J.;Arganda-Carreras, I.;Frise, E.;Kaynig, V.;Longair, M.;Pietzsch, T., *et al.* Fiji: an open-source platform for biological-image analysis. *Nat Methods*. **2012**;*9*(7):676-82. doi:10.1038/nmeth.2019.
8. Miller, J.L.;Watkin, K.L.Chen, M.F. Muscle, adipose, and connective tissue variations in intrinsic musculature of the adult human tongue. *J Speech Lang Hear Res*. **2002**;*45*(1):51-65. doi:10.1044/1092-4388(2002/004).
9. Pfaffl, M.W. A new mathematical model for relative quantification in real-time RT-PCR. *Nucleic Acids Res*. **2001**;*29*(9):e45. doi:10.1093/nar/29.9.e45.

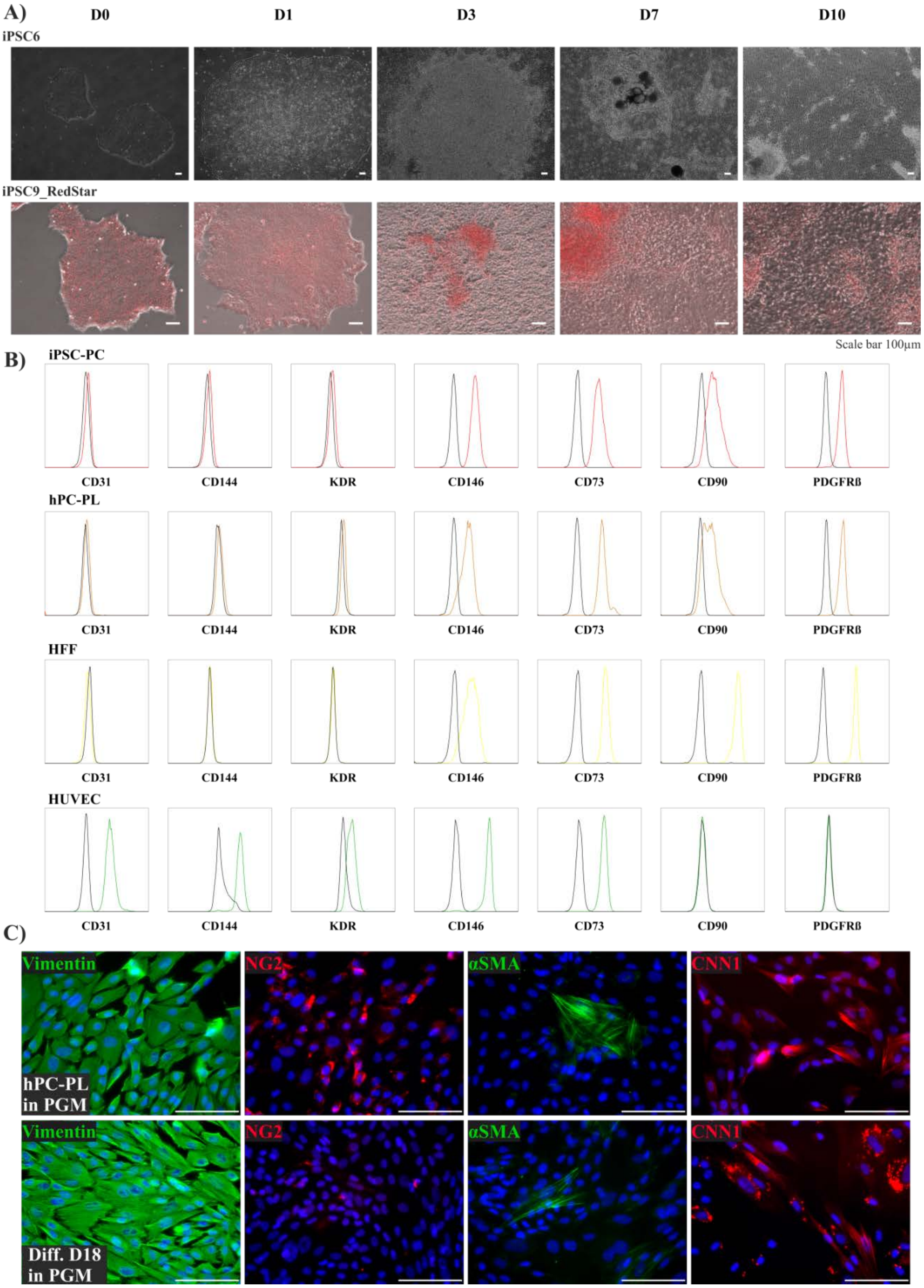

Figure S.1. – Differentiation of PC-like cells

(A) Morphological changes over the first 10 days of differentiation were monitored in the iPSC6 cells and in the iPSC9_RedStar cells. (B) Endothelial and mesenchymal surface marker expression profile of human differentiated (iPSC-PC) and primary PCs (hPC-PL), dermal fibroblasts (hFF), and umbilical vein ECs (HUVEC). (C) Immunofluorescent staining for vimentin (VIM), NG2, α-smooth muscle actin (αSMA) and calponin-1 (CNN1) in hPC-PLs and iPSC-PCs on D18 of differentiation. Scale bars: 100 µm.

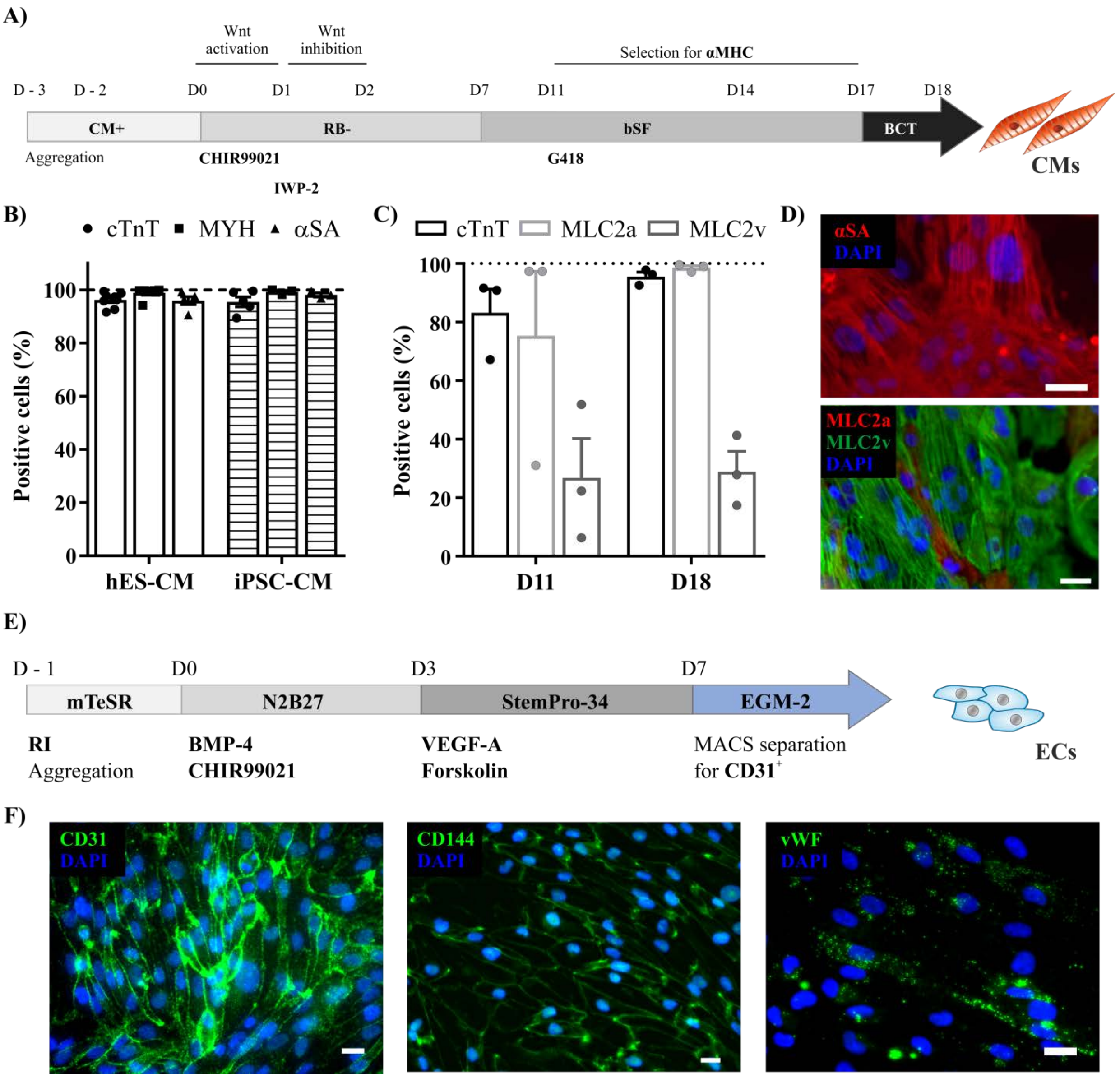

Figure S.2. – Differentiation of CMs and ECs

Timeline of the cardiac differentiation process (A). Culture media used for the differentiation are labelled on the arrow. (B) CM marker (cTnT, MYH, αSA) expression after selection in hESC and iPSC-derived CMs (n = 3-8). (C) Differentiation efficiency and CM-subtype markers (MLC2a and MLC2v) before (D11) and after selection (D18) (hESC-CMs, n =3). (D) D40 hESC-CMs, seeded on fibronectin-gelatin coated plates, stained for αSA, MLC2a, and MLC2v, demonstrated a ventricular specification. (E) Differentiation protocol towards ECs. (F) Expression of the EC-markers CD31, CD144 and von Willebrand factor (vWF) in CD31-sorted iPSC-ECs. Scale bar: 20µm.

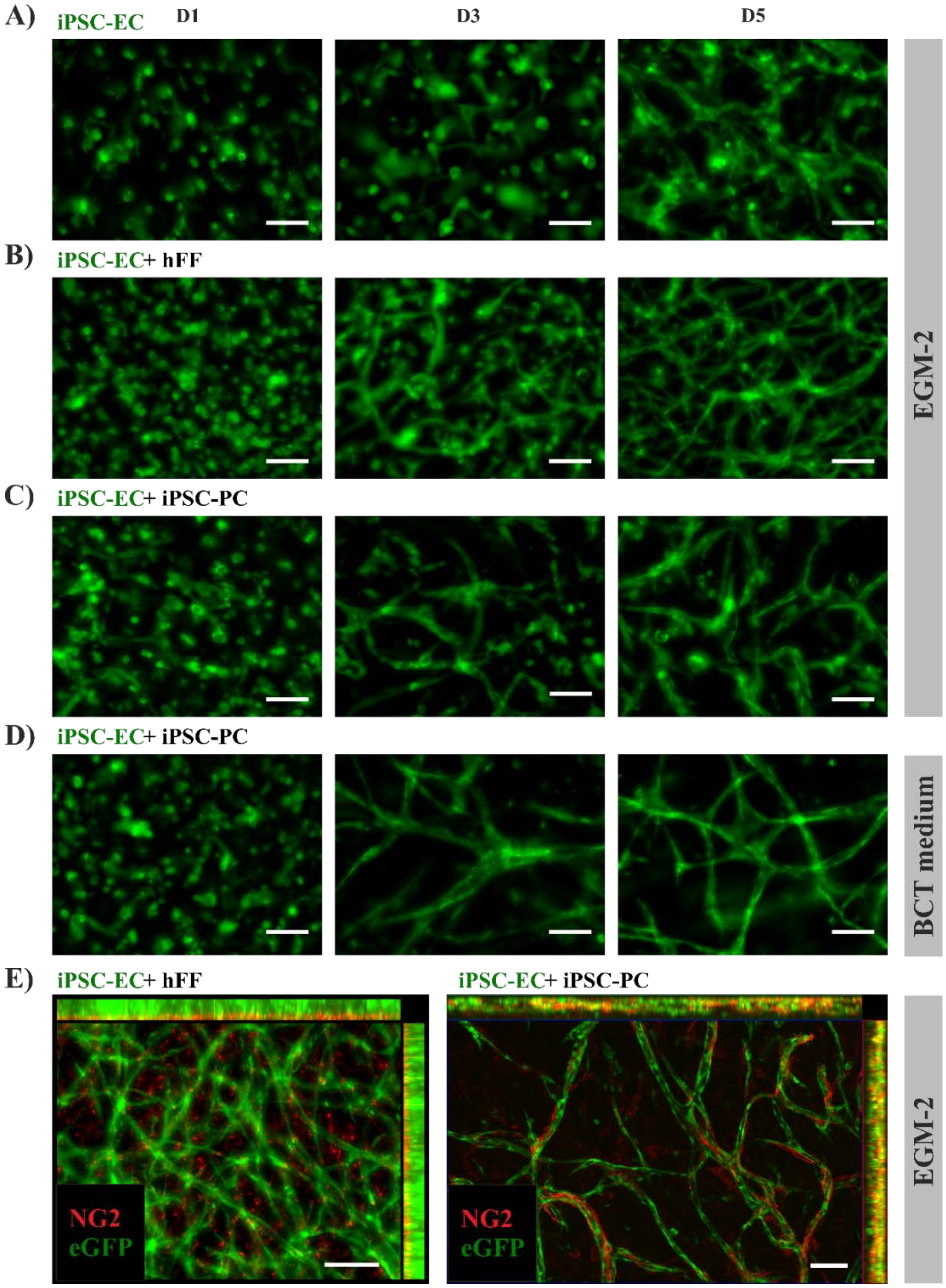

Figure S.3. – Co-culture of iPSC-PCs or hFFs with iPSC-ECs in fibrin gels

Live imaging of different cell compositions encapsulated in fibrin matrices on day 1, 3 and 5 of cultivation: iPSC-ECs alone in endothelial cell medium EGM-2 (A), iPSC-EC+hFF in EGM-2 (B), and iPSC-EC+iPSC-PC both in EGM-2 (C) and BCT medium (D). The iPSC-ECs express endogenous eGFP, other cell types were not labelled. (E) Maximum intensity projection images with orthogonal view showing immunostained (red - NG2) co-cultures fixed on D7. NG2^{+}cells were forming a single layer below EC-networks in the iPSC-EC+hFF group, whereas in the iPSC-EC+iPSC-PC group the NG2+ cells are located in close proximity to ECs. Scale bar: 100 µm.

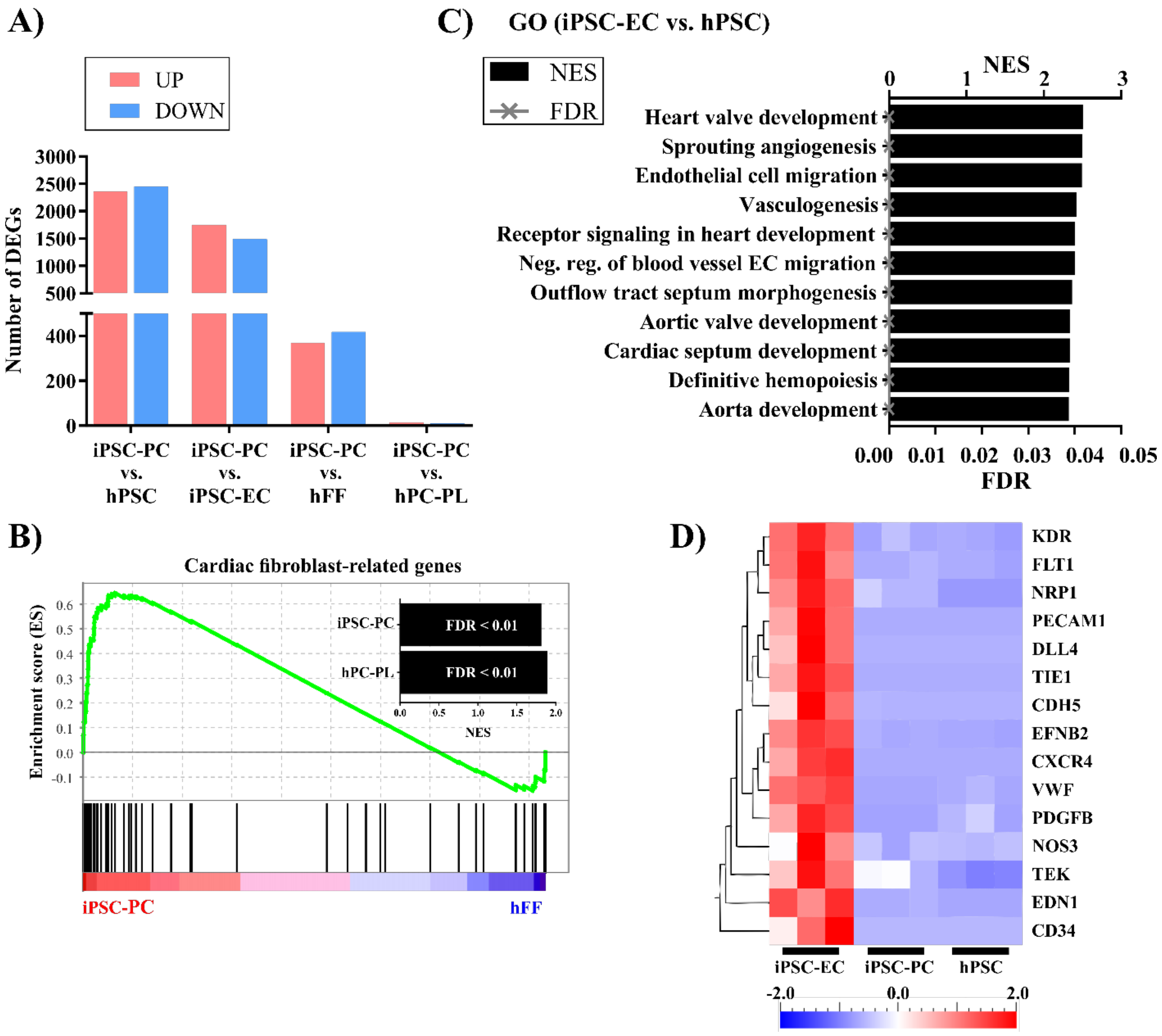

Figure S.4. – Characterisation of differentiated vascular cells

(A) Differentially expressed genes (DEG) between iPSC-PCs and the other samples (fold change ≥ 2, FDR ≤ 0.05). (B) Enrichment plot (iPSC-PCs vs. hFF) including genes upregulated in cardiac fibroblast transcriptome. Additionally, NES values for both hPC-PL and iPSC-PC are showed. (C) Normalized enrichment scores (NES) of significantly enriched GOs iPSC-EC vs. hPSC. (D) Hierarchical clustering of EC-related genes expressed in iPSC-ECs, iPSC-PCs and hPSCs.

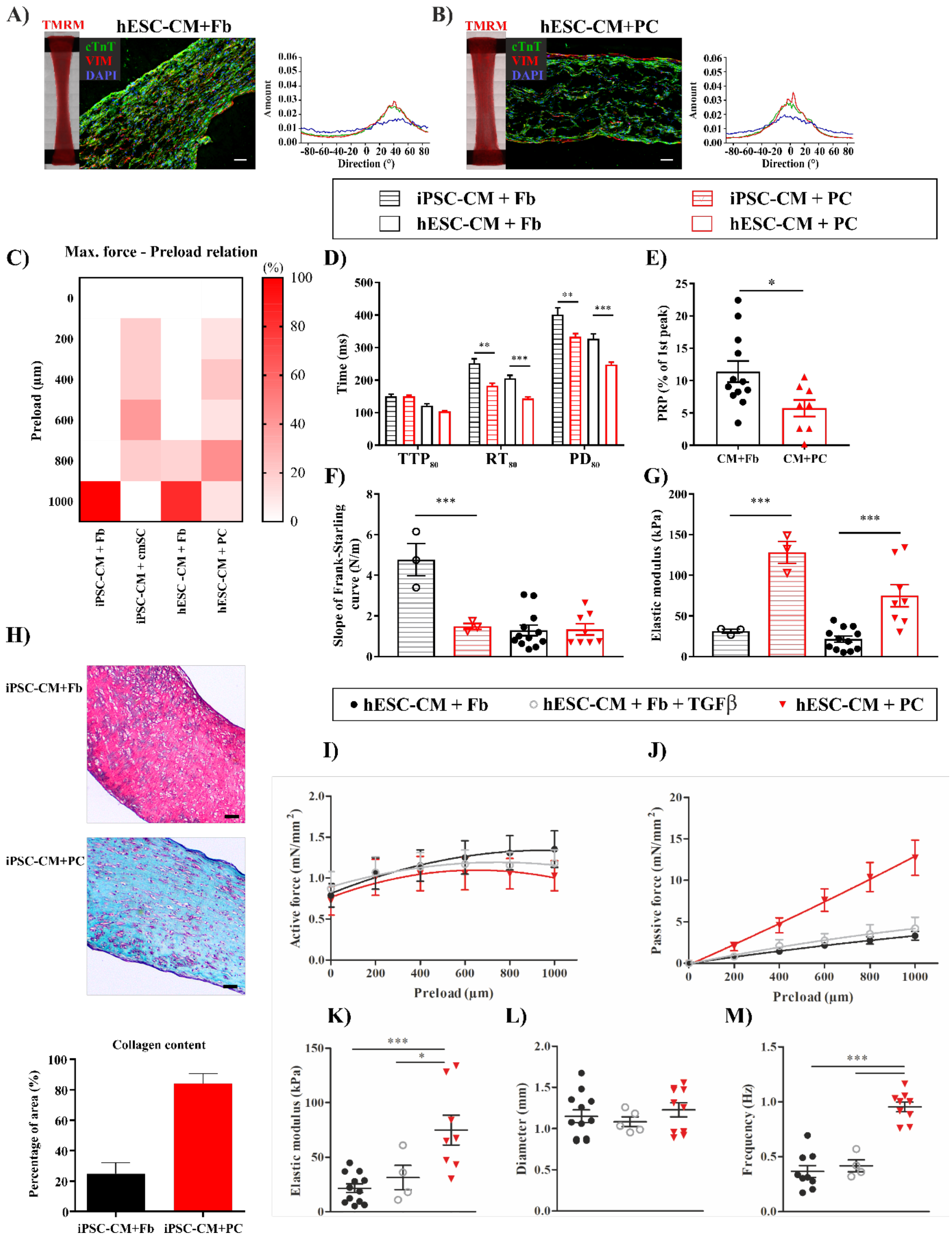

Figure S.5. - Effect of iPSC-PCs and TGFβ on electromechanical properties of BCTs

(A) hES-CM+Fb and (B) hES-CM+PC live tissue morphology on D21 (CMs visualized with TMRM) and fixed slices stained for cTnT and vimentin (nuclei: DAPI). Plots of directionality analysis depict the 3 fluorescent channels, where the Y-axis label 'Amount' estimates the proportion of structures with the dominant orientation (C) Heatmap describing the percentage of BCTs reaching maximum active force, indicating optimal sarcomere length, at each preload

*step. (D) Time to peak (TTP), relaxation time (RT), and peak duration (PD) measured at 80% peak height. (E) Post-rest potentiation (PRP) given as percentage of the first peak following high frequency pacing in BCTs with hESC-CMs. (F) Maximum slope of the Frank-Starling curves. (G) Elastic modulus calculated from the stress-strain curve (n = 9-12 BCTs per group with hESC-CM and n = 3-5 with iPSC-CM, ** $p<0.01$ *** $p<0.001$). (H) Gömöri's Trichrome staining for visualization and quantification of collagen content (blue area measured; n=2 BCTs per group). Tissue properties on D21 following 7 days of TGFβ treatment compared to non-treated hESC-CM based BCTs (I-M). (I) Active isometric contraction forces. (J) Passive forces. (K) Elastic moduli. (L) Tissue diameter. (M) Spontaneous contraction frequency (n = 4-12 BCTs, * $p<0.05$ *** $p<0.001$). (Scale bars: 100 µm).*

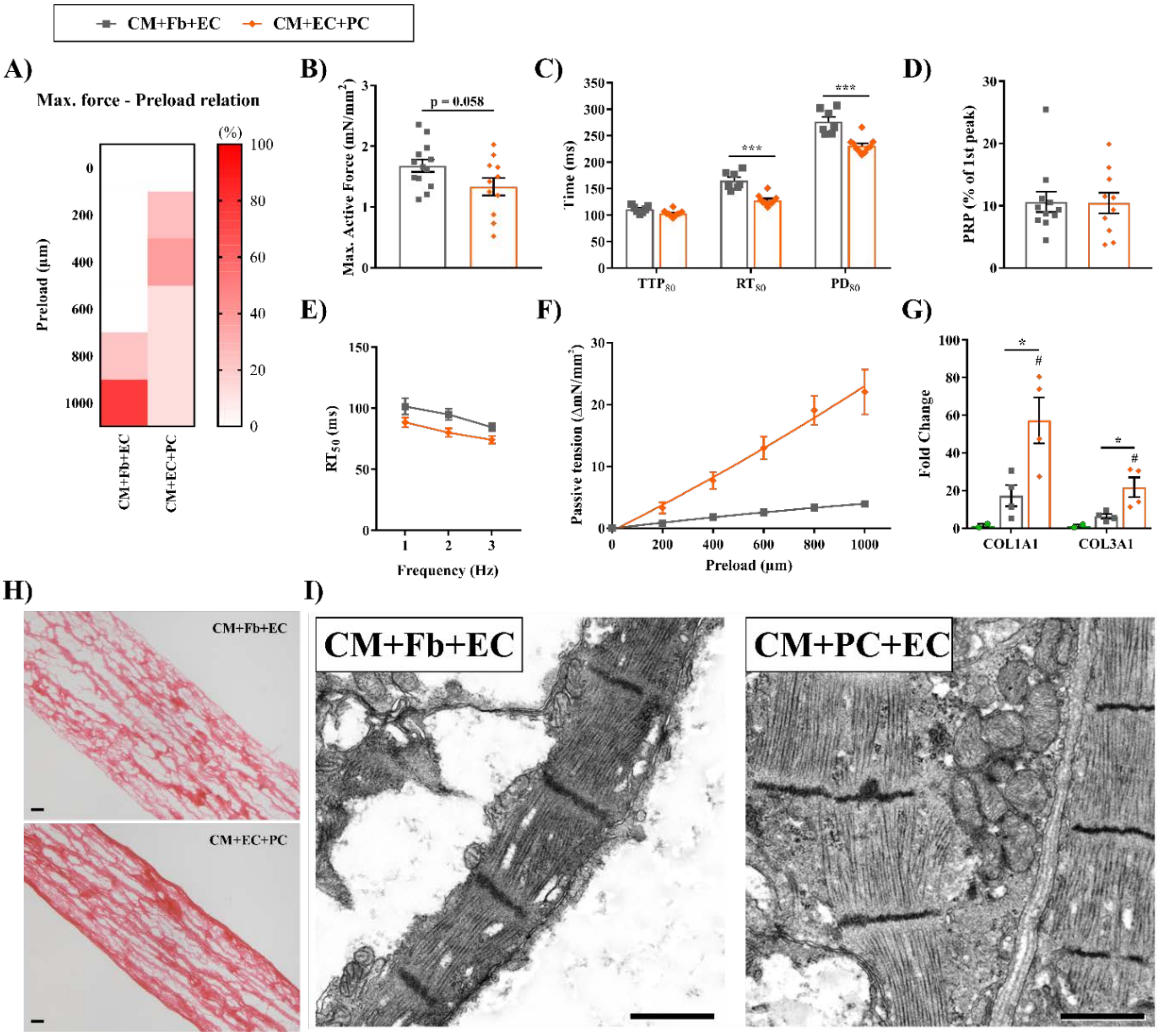

Figure S.6. – Properties of EC-containing BCTs

(A) *Heatmap describing the percentage of BCTs reaching maximum active force, indicating optimal sarcomere length, at each preload step. (B) Maximum active force values measured at optimal sarcomere length (L_{max}). (C) Time to peak (TTP), relaxation time (RT), and peak duration (PD) measured at 80% peak height. (D) Post-rest potentiation (PRP) given as percentage of the first peak following high frequency pacing. (E) Relaxation time/frequency relationship. (F) Passive tension development with increasing preload. (n = 5-16 BCTs/group; *p<0.05 ***p<0.001). (G) Expression of COL1A1 and COL3A1 determined by qRT-PCR (Human ventricle was used as control-green, the expression is relative to GAPDH; n = 3-4 tissues; *p<0.05 **p<0.01 between indicated groups and # p<0.05 vs. human ventricle). (H) Sirius Red staining showing collagen distribution within BCT sections. Scale bars: 100 µm. (I) Details of myofibrils by Transmission Electron Microscopy of longitudinally sectioned EC-containing BCTs. Left: Myosin filaments are oriented parallel inside the myofibril, Z-bands are regular and straight. Right: Myofibrils (including Z-bands) are wider than in the other tissues and are also more frequently detectable. Scale bars: 1 µm.*

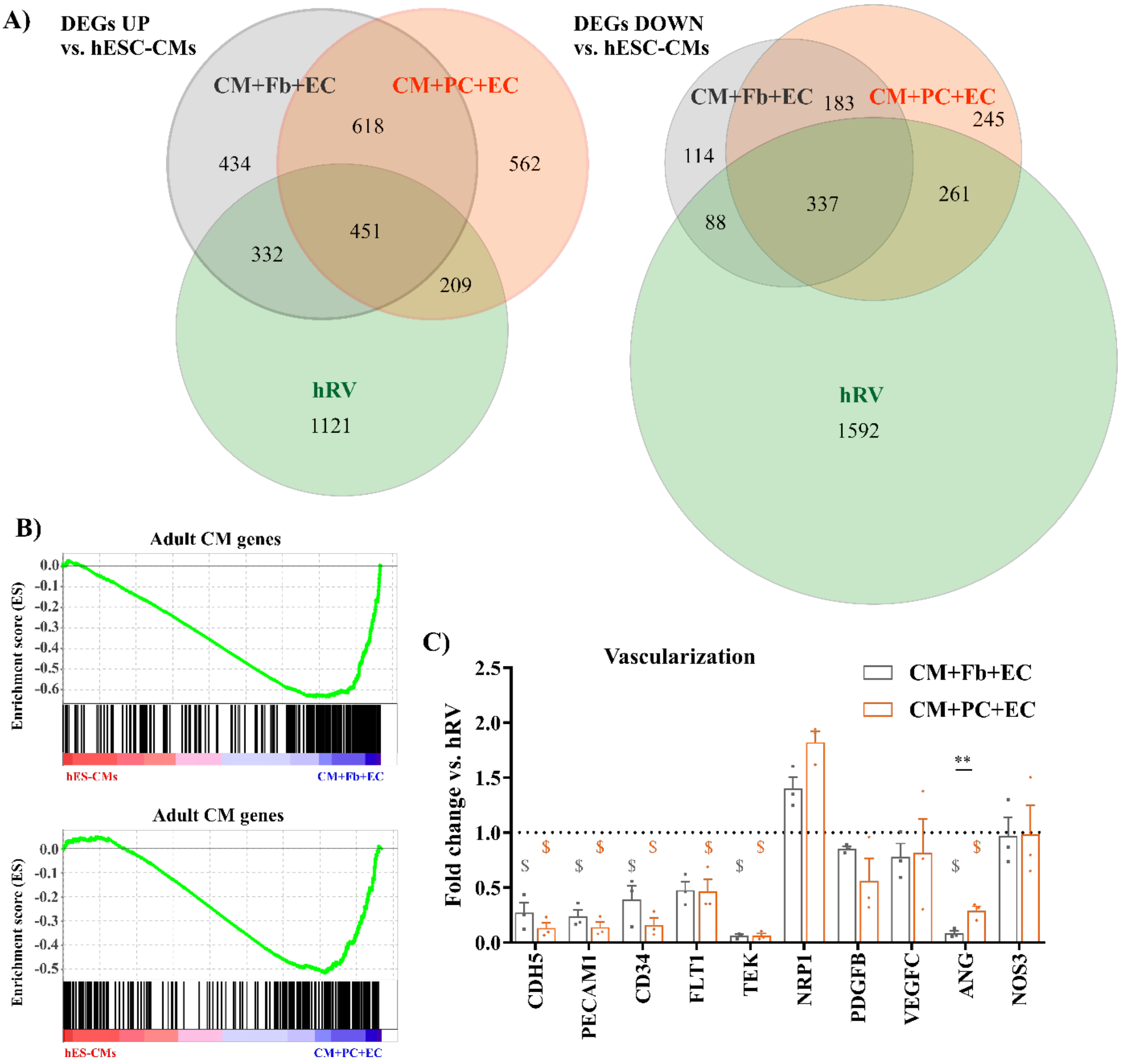

Figure S.7. – Gene expression profile of multi cell type BCTs

*(A) Number and distribution of differentially expressed genes (DEG) across human right ventricle (hRV) and BCT types vs. hESC-CMs (FC≥2 and FDR≤0.05). (B) GSEA analysis of hESC-CMs vs. BCT-types using a gene set for adult CM-associated genes (218 genes). (C) Expression of angiogenesis related genes in BCTs normalized to hRV (Genes marked with $ are significant vs. hRV; ** p<0.01between indicated groups, n=3).*
